# A Novel One-Pot Green Synthesis of Dispirooxindolo-pyrrolidines via1,3-Dipolar Cycloaddition Reactions of Azomethine Ylides

**DOI:** 10.3390/molecules20010780

**Published:** 2015-01-07

**Authors:** Abdulrahman I. Almansour, Natarajan Arumugam, Raju Suresh Kumar, Govindasami Periyasami, Hazem A. Ghabbour, Hoong-Kun Fun

**Affiliations:** 1 Department of Chemistry, College of Science, King Saud University, P.O. Box 2455, Riyadh 11451, Saudi Arabia; 2 Centro de Química-Física Molecular and Institute of Nanoscience and Nanotechnology, Instituto Superior Técnico, Universidade de Lisboa, Lisboa 1049-001, Portugal; 3 Department of Pharmaceutical Chemistry, College of Pharmacy, King Saud University, P.O. Box 2457, Riyadh 11451, Saudi Arabia; 4 X-ray Crystallography Unit, School of Physics, Universiti Sains Malaysia, Penang 11800, Malaysia

**Keywords:** 1,3-dipolar cycloaddition, azomethine ylide, dispiropyrrolidines, ionic liquids

## Abstract

A facile synthesis of dispirooxindolopyrrolidines has been accomplished via a one-pot three component 1,3-dipolar cycloaddition reaction. The reaction of azomethine ylides generated *in situ* from L-phenylalanine and substituted isatins with a series of unusual (*E*)-2-oxoindolino-3-ylidene acetophenone dipolarophiles in the ionic liquid 1-butyl-3-methylimidazolium bromide [bmim]BF_4_, furnished the cycloadducts in good yields, with the regioisomers **5a**–**f** being obtained with high selectivity. Furthermore, the recyclability of [bmim]BF_4_, up to five times, was also investigated.

## 1. Introduction

Ionic liquids are widely recognized as “green” solvents in organic synthesis because of their unique properties, such as low vapor pressure, high chemical and thermal stability, good solvating ability, non-flammability, behavior as acidic or basic catalysts and recyclability [1,2,3,4,5]. In this context, ionic liquids have emerged as new green solvents to replace volatile organic compounds and they are suitable for executing many diverse organic reactions [6,7,8]. One-pot three component reactions, widely useful in both combinatorial and medicinal chemistry arenas, with their powerful bond forming and atom efficiencies, represent another eco-friendly synthetic approach for the expedient construction of molecules of structural diversity and complexity [9,10,11,12]. These reactions also assume importance from the viewpoint of minimized waste generation due to the avoidance of intermediate isolation and purification steps. For these reasons, the development of multi-component reactions in ionic liquids, although relatively unexplored [13], is of great interest.

**Figure 1:**
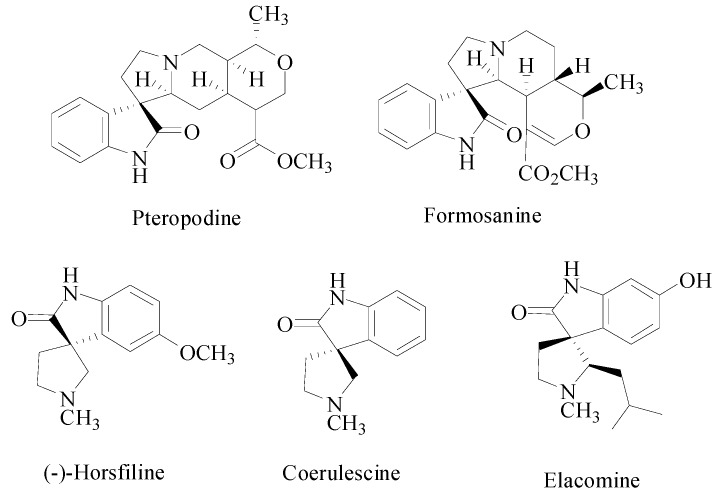
Biologically relevant spirooxindolopyrrolidine alkaloids and natural products.

The spirooxindole core is a privileged heterocyclic system that is featured in a large number of bioactive naturally occurring alkaloids (Figure 1) that display a wide spectrum of biological activities such as antimicrobial [14], inhibition of human NK-1 receptor [15] and potent non-peptide inhibition of the p53–MDM2 interaction [16]. Particularly, the spiropyrrolidinyloxindole nucleus is often found in the molecular framework of many natural products, *viz*., horsfiline [17], coerulescine [18], and elacomine [19] (Figure 1) which possess a myriad of biological activities such as inhibition of the mammalian cell cycle at G2/Mphase [20,21], inhibition of microtubule assembly [22], modulation of the function of muscarinic serotonin receptors [23], antitumor activity against human brain cancer cell lines, neuroblastoma SKN-BE (2), and malignant glioma GAMG [24].

The biological significance of the above heterocycles prompted us to explore the synthesis of novel hybrid spiroheterocycles comprising pyrrolidine and oxindole units via a one-pot three component 1,3-dipolar cycloaddition reaction. Recently, we embarked on a program for the synthesis and/or biological screening of structurally diverse novel spiroheterocycles, which has brought to light various antimicrobial leads [25,26,27,28]. In continuation of our research in the area of 1,3-dipolar cycloaddition reactions [29,30], herein we wish to report an expeditious and facile protocol for the synthesis of dispirooxindolopyrrolidines in ionic liquid medium; highly desirable from the viewpoint of green chemistry. Furthermore, to the best of our knowledge, this is the first report on the generation of azomethine ylide from L-phenylalanine and isatin in ionic liquid medium.

## 2. Results and Discussion

**Scheme 1:**
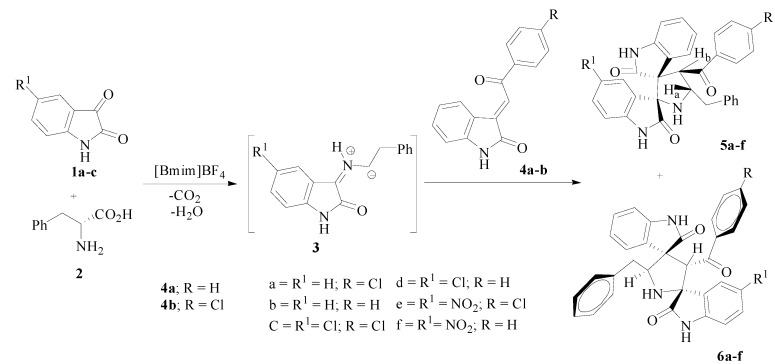
Synthesis of dispirooxindolopyrrolidines **5a**–**f** and **6a**–**f**.

In the present investigation, the one-pot three component reaction of (*E*)-2-oxoindolino-3-ylidene acetophenones **4a**–**b** with non-stabilized azomethine ylides **3**, generated *in situ* by the decarboxylative condensation of isatin **1a**–**c** and L-phenylalanine (**2**) in [bmim]BF_4_ afforded the dispiropyrrolidines **5a**–**f** in good yields (70%–77%) with selectivity, along with trace amounts of the regioisomers **6a**–**f** (5%–7%) (Scheme 1).

Solvent-optimization for this cycloaddition reaction was investigated by the reaction of an equimolar ratio of (*E*)-2-oxoindolino-3-ylidene acetophenone, isatin and L-phenylalanine in organic solvents *viz*. methanol, ethanol, dioxane, dioxane/methanol (1:1) mixture under heating in an oil-bath (Table 1). The cycloadducts **5a** and **6a** were obtained only in 28%and 12% yield in methanol (Table 1, entry 1), whilst the reactions in ethanol, dioxane and dioxane/methanol (1:1) mixture furnished the products **5a** and **6a** in 30% and 15%, 34% and 16%, 38% and 18% yields, respectively, which indicated that the solvents had little effect on the selectivity and yield of the reaction (Table 1). The same reaction was also investigated in ionic liquids, such as [bmim]Br, [bmim]BF_4_ and also with combination of a catalyst, [bmim][BF_4_]/CuI (10 mol %), [bmim][BF_4_]/Zn(OTf)_2_ (10 mol %). Like the reactions in organic solvents, these reactions also furnished **5a** and **6a**, but with good yield and high selectivity, the isomer **5** being obtained predominantly in all cases. The ionic liquids [bmim]BF_4_ and [bmim]Br were found to be the appropriate reaction medium for these cycloaddition reactions in terms of yield and selectivity (Table 1, entry 6). Hence, all the subsequent reactions were performed by heating an equimolar mixture of the reactants in [bmim]BF_4_ (3 mL) in an oil-bath at 100 °C for 2 h (Table 2). After completion of the reaction (by TLC), the product was isolated and purified by flash column chromatography, while the [bmim]BF_4_was recovered by vacuum distillation then dried under vacuum at 40 °C overnight for recycling (Table 3).

**Table 1:** Solvent condition and yield optimization of cycloaddition reaction.

Entry	Solvent System	Yield (%)		Time (h)
		5a	6a	
1	Methanol	28	12	6
2	Ethanol	30	15	6
3	Dry Dioxane	34	16	5
4	Dioxane/Methanol	38	18	4
5	[bmim]Br	69	8	2
6	[bmim][BF_4_]	77	7	2
7	[bmim][BF_4_]/CuI (10 mol %)	60	15	2
8	[bmim][BF_4_]/Zn(OTf)_2_ (10 mol %)	62	18	2

Notes: Optimized reaction condition is in bold (entry 6); Ionic liquids were subjected to high vacuum before use (entry 5–8).

**Table 2:** Yield and distereoselectivity of the cycloadducts **5a**–**f** and **6a**–**f**.

Entry	Derivatives (a–f)	Yield of the Cycloadducts (%) ^#^		Diastereoselectivity (5/6)
		5	6	
1	**a**	77	7	91/9
2	**b**	70	5	85/15
3	**c**	75	6	89/11
4	**d**	73	5	90/10
5	**e**	72	5	88/12
6	**f**	71	5	87/13

Note: ^#^ All reactions were carried out with [bmim]BF_4_ ionic liquid under 2 h refluxion.

**Table 3:** Reusability of the ionic liquid in the synthesis of **5a** and **6a**.

Experiment	First	Second	Third	Fourth	Fifth
[Bmim]Br(Yield %)	69:8	67:7	63:6	61:4	60:3
[Bmim]BF_4_(Yield %)	77:7	75:6	72:5	68:4	65:3

**Figure 2:**
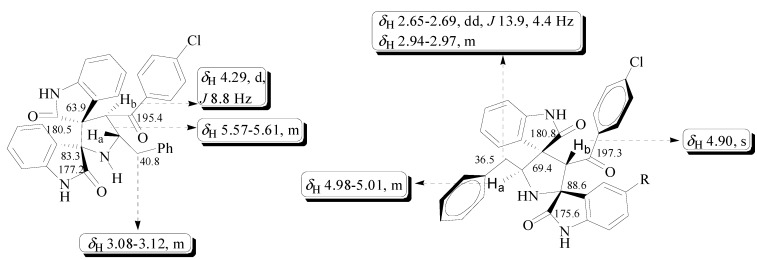
Selected ^1^H- and ^13^C-NMR signals of compounds **5a** and **6a**.

The structure of regioisomers was elucidated using IR, ^1^H-, ^13^C-NMR spectroscopic and mass spectrometry studies. For instance, the IR spectrum of the cycloadduct **5a** displayed characteristic bands at 1628, 1706, 1710 cm^−1^ corresponding to the two oxindoles and benzoyl carbonyls, respectively. In the ^1^H-NMR spectrum of **5a**, the benzylic and H_a_ protons appeared as multiplets in the region δ_H_ 3.08–3.12 and 5.57–5.61 ppm, respectively. The H_b_ proton attached to the benzoyl group appeared as doublet at δ_H_ 4.29 ppm and the *trans* stereochemistry was confirmed through coupling constant of H_b_ proton (*J* = 8.8 Hz). The aromatic protons appear as multiplets around 6.43–7.78 ppm. In the ^13^C-NMR spectrum, the signals at δ_C_ 177.2, 180.5 and 195.4 ppm were attributed to two oxindole and benzoyl ring carbonyls. The two spiro carbons resonated at δ_C_ 63.9 and 83.3 ppm. Furthermore, the presence of the molecular ion peak at *m*/*z* 533 (M^+^) in the mass spectrum of **5a** supported the formation of the cycloadduct. The stereochemistry of compound **5a** has been assigned based on the fact that the carbonyls of the two isatin rings are in *trans* relationship as to minimize the repulsion between the two isatin carbonyl as evidenced in the literature [31,32,33]. The structure of other regioisomer **6a** was also elucidated by similar straight forward considerations. The singlet at δ_H_ 4.90 ppm in the ^1^H-NMR spectrum, confirms the formation of regioisomer **6a**. A pictorial representation of the ^1^H- and ^13^C-NMR chemical shifts of **5a** and **6a** is shown in Figure 2.

**Scheme 2:**
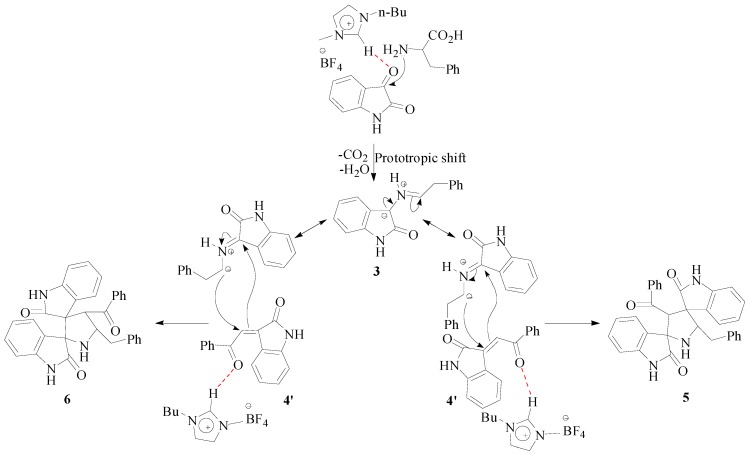
Plausible mechanism for the synthesis of dispirooxindolopyrrolidine regioisomers.

A rational mechanism for the formation of the cycloadducts **5** and **6** in the presence of [bmim]BF_4_ is described in Scheme 2. It is known that ionic liquid play dual roles as solvent and catalyst. For instance, the hydrogen atom of [bmim]^+^, being electron-deficient can form hydrogen bonds with heteroatoms thereby catalyzing reactions. Thus, in the present transformation, a hydrogen bond between the imidazole ring hydrogen atom of [bmim]^+^ and the carbonyl group of **4** furnishes **4**', which readily reacts with azomethine ylide to afford **5** and **6**. The azomethine ylide may also be catalyzed by [bmim]BF_4_ via hydrogen bonding. This catalysis presumably expedites the reaction in ionic liquid relative to other solvents.

**Figure 3:**
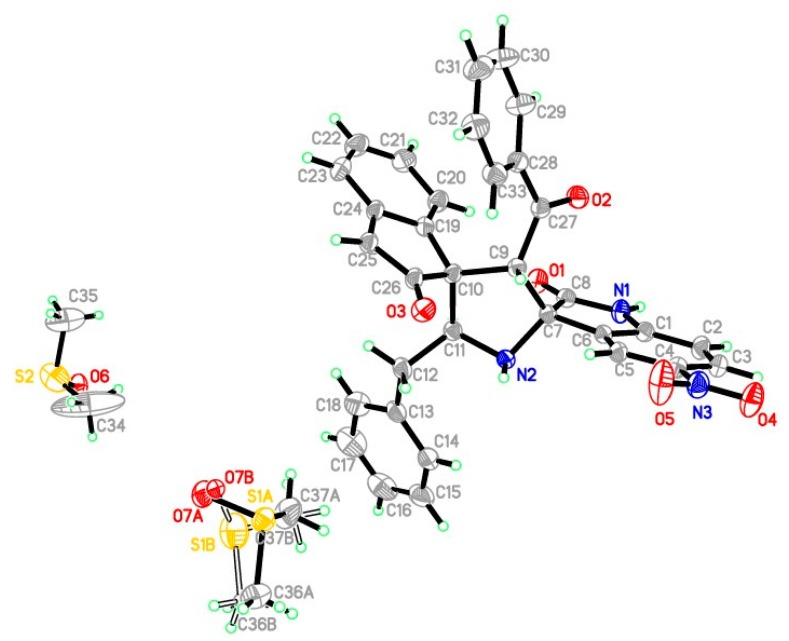
ORTEP diagram of **6f**, showing the atom-numbering. Displacement ellipsoids are drawn at the 40% probability level and all *H*-atoms are shown as small spheres of arbitrary radii.

The regio- and stereo- chemistry of cycloadduct **6f** was further supported by single crystal X-ray diffraction studies [34] (Figure 3). The molecule **6f** is composed of a central pyrrolidine ring with benzoyl group at C9, benzyl group at C11, spiro-carbons fused with oxindoles at C7 and C10 (Figure 3). The dihedral angle between the pyrrolidine ring A (N2/C7/C9/C10/C11), indolin-2-one ring B (C1-C8/N1) and 2,3-dihydro-1*H*-inden-1-one ring C (C19-C26) are 77.96(2)°, 84.72(3)° and 24.07(2)° for A/B, A/C and B/C, respectively. The torsion angle in between C11-N2-C7-C9 is 40.0(2)°. There are three intramolecular interactions between C9–H9A···O3, C11–H11A···O1 and C20–H20A···O1. In the crystal structure, seven intermolecular N–H···O and C–H···O hydrogen bonds are observed. The selected geometric parameters and distances of the donor–H, acceptor/H, donor/acceptor and donor–H/acceptor angles are presented in Tables S1 and S2 (*vide* Supplementary Data).

## 3. Experimental Section

### 3.1. General Methods

Melting points were taken using open capillary tubes and are uncorrected. Unless stated otherwise, solvents and chemicals were obtained from commercial sources and used without further purification. IR Spectra were measured as KBr pellets on a Nicolet 6700 FT-IR spectrophotometer (Madison, WI, USA). ^1^H- and ^13^C-NMR spectra were recorded on a Varian Mercury JEOL-400 NMR spectrometer (Tokyo, Japan) and Bruker 500 MHz NMR spectrometers (Faellanden, Switzerland) operating at 400, 500, 100 and 125 MHz, respectively, and chemical shifts are reported as δ values (ppm) relative to tetramethylsilane. Elemental analyses were performed on a Perkin-Elmer 2400 series II elemental CHNS analyser (Waltham, MA, USA). Flash column chromatography was performed on silica gel (230–400 mesh) using petroleum ether (60–80 °C)/EtOAc as eluent.

### 3.2. General Procedure for Synthesis of Dispirooxindoles **5a**–**f** and **6a**–**f**

A mixture of isatin (1 mmol), L-phenylanaine (1 mmol) and (*E*)-2-oxoindolino-3-ylidene acetophenone (1 mmol) was heated with stirring in [bmim]BF_4_ medium (3 mL) for 2 h at 100 °C. After completion of the reaction as evidenced by TLC analysis, ethyl acetate (10 mL) was added and the reaction mixture was stirred for 10–15 min. The organic layer was separated and removed under reduced pressure. The crude cycloadducts were isolated through flash column chromatography. The ionic liquid [bmim]BF_4_ after extraction of the product was dried under vacuum at 40 °C for 2 h overnight to eliminate any water trapped from moisture and reused for subsequent runs.

*(2′R,3′R,4′S,5′S)-4′-(4-Chlorobenzoyl)-5′-benzyl-spiro[3,2′]oxindolo-spiro[3,3″]oxindolopyrrolidine* (**5a**). Colorless solid (290 mg, 77%), mp 195–197 °C (EtOAc); *R*_F_ 0.4 (pet. ether/EtOAc, 1:1); IR (KBr): 1628, 1706 and 1710 cm^−1^; ^1^H-NMR (400 MHz, CDCl_3_): δ_H_ 7.78–6.43 (m, 17H, aromatic), 5.61–5.57 (m, 1H), 4.29 (d, *J* = 8.8 Hz, 1H), 3.12–3.08 (m, 2H); ^13^C-NMR (100 MHz, CDCl_3_): δ_C_ 195.4, 180.5, 177.2, 141.3, 140.3, 138.8, 138.2, 135.9, 130.0, 129.8, 129.4, 128.9, 128.8, 128.5, 127.7, 126.6, 125.6, 125.3, 124.3, 122.6, 109.5, 109.3, 83.3, 63.9, 61.4, 59.5, 40.8. LC/MS (ESI): *m*/*z* 533 [M]^+^. Anal. Calcd for: C_32_H_24_ClN_3_O_3_: C, 71.97; H, 4.53; N, 7.87. Found: C, 71.81; H, 4.68; N, 7.93%.

*(2′S,3′S,4′S,5′S)-3′-(4-Chlorobenzoyl)-5′-benzyl-spiro[3,2′]oxindolo-spiro[3″,4′]oxindolopyrrolidine* (**6a**). Colorless solid (26 mg, 7%), mp 162–164 °C (EtOAc); *R*_F_ 0.5 (pet. ether/EtOAc, 1:1); IR (KBr): 1626, 1708 and 1710 cm^−1^; ^1^H-NMR (400 MHz, CDCl_3_): δ_H_ 7.69–6.37 (m, 17H, aromatic), 5.01–4.98 (m, 1H), 4.90 (s, 1H), 2.97–2.94 (m, 1H), 2.69–2.65 (dd, *J* = 13.9, 4.4 Hz, 1H); ^13^C-NMR (100 MHz, CDCl_3_): δ_C_ 197.3, 180.8, 175.6, 141.3, 140.2, 139.6, 135.5, 131.3, 129.5, 128.8, 128.6, 128.4, 128.4, 128.4, 128.2, 127.8, 126.3, 125.3, 124.8, 122.6, 110.0, 109.8, 88.6, 69.4, 68.6, 61.6, 36.5. LC/MS (ESI): *m*/*z* 533 [M]^+^. Anal. Calcd for: C_32_H_24_ClN_3_O_3_: C, 71.97; H, 4.53; N, 7.87. Found: C, 71.88; H, 4.65; N, 7.96%.

*(2′R,3′R,4′S,5′S)-4′-Benzoyl-5′-benzyl-spiro[3,2′]oxindolo-spiro[3,3″]oxindolopyrrolidine* (**5b**). Color-less solid (281 mg, 70%), mp 145–147 °C (EtOAc); *R*_F_ 0.4 (pet. ether/EtOAc, 1:1); IR (KBr): 1628, 1706 and 1710 cm^−1^; ^1^H-NMR (500 MHz, CDCl_3_): δ_H_ 6.31–7.42 (m, 16H, aromatic), 5.56–5.54 (m, 1H), 4.30 (d, *J* = 8.8 Hz, 1H), 3.04–3.02 (m, 2H); ^13^C-NMR (125 MHz, CDCl_3_): δ_C_ 196.7, 181.0, 177.7, 177.7, 141.5, 140.5, 138.3, 137.6, 132.5, 129.9, 129.4, 128.7, 128.6, 128.6, 128.4, 128.2, 127.5, 126.4, 125.5, 125.4, 124.4, 122.4, 109.6, 109.2, 83.9, 63.9, 61.2, 59.5, 40.7. LC/MS (ESI) *m*/*z* 499 [M]^+^; Anal. Calcd for: C_32_H_25_N_3_O_3_: C, 76.94; H, 5.04; N, 8.41. Found: C, 76.88; H, 5.16; N, 8.52%.

*(2′S,3′S,4′S,5′S)-3′-Benzoyl-5′-benzyl-spiro[3,2′]oxindolo-spiro[3″,4′]oxindolopyrrolidine* (**6b**). Color-less solid (20 mg, 5%), mp 117–119 °C (EtOAc); *R*_F_ 0.5 (pet. ether/EtOAc, 1:1); IR (KBr): 1628, 1706 and 1710 cm^−1^; ^1^H-NMR (500 MHz, CDCl_3_): δ_H_ 7.48–6.26 (m, 18H, aromatic), 5.39–5.37 (m, 1H), 4.88 (s, 1H), 3.48–3.47 (m, 1H), 3.17–3.16 (m, 1H); ^13^C-NMR (125 MHz, CDCl_3_): δ_C_ 197.9, 179.8, 174.6, 142.2, 141.1, 139.8, 137.3, 132.5, 129.5, 129.1, 128.9, 128.2, 128.4, 128.3, 128.1, 127.5, 126.3, 125.8, 125.5, 121.1, 109.6, 109.5, 75.2, 67.1, 64.8, 61.3, 39.7. LC/MS (ESI) *m/z* 499 [M]^+^; Anal. Calcd for: C_32_H_24_N_3_O_3_: C, 76.94; H, 5.04; N, 8.41. Found: C, 76.85; H, 5.21; N, 8.54%.

*(2′R,3′R,4′S,5′S)-4′-(4-Chlorobenzoyl)-5′-benzyl-5-chlorospiro[3,2′]oxindolo-spiro[3,3″]oxindolo-pyrrolidine* (**5c**). Pale yellow solid (301 mg, 75%), mp 205–207 °C (EtOAc); *R*_F_ 0.4 (Pet. Ether/EtOAc, 1:1); IR (KBr): 1625, 1708 and 1710 cm^−1^; ^1^H-NMR (400 MHz, CDCl_3_): δ_H_ 8.31 (s, 1H, NH), 8.29 (s, 1H, NH), 7.48–6.45 (m, 16H, aromatic), 5.57–5.51 (m, 1H), 4.26 (d, *J* = 8.1, 1H), 3.08–3.03 (m, 2H); ^13^C-NMR (100 MHz, CDCl_3_): δ_C_ 195.3, 177.2, 140.5, 140.2, 138.2, 139.0, 135.8, 130.0, 129.3, 129.0, 128.9, 128.5, 127.9, 127.6, 127.1, 126.6, 126.1, 124.0, 122.7, 110.8, 109.6, 83.0, 63.7, 61.5, 59.4, 41.0. LC/MS (ESI) *m*/*z* 567 [M]^+^; Anal. Calcd. for: C_32_H_23_Cl_2_N_3_O_3_: C, 67.61; H, 4.08; N, 7.39. Found: C, 67.76; H, 4.19; N, 7.47%.

*(2′S,3′S,4′S,5′S)-3′-(4-Chlorobenzoyl-5′-benzyl)-5-chlorospiro[3,2′]oxindolo-spiro[3″,4′]oxindolo-pyrrolidine* (**6c**). Pale yellow solid (24 mg, 6%), mp 187–189 °C (EtOAc); *R*_F_ 0.5 (Pet. Ether/EtOAc, 1:1); IR (KBr): 1625, 1708 and 1710 cm^−1^; ^1^H-NMR (400 MHz, CDCl_3_): δ_H_ 8.71 (s, 1H, NH), 8.53 (s, 1H, NH), 5.16 (s, 1H), 7.66–6.29 (m, 16H, aromatic), 5.00–4.96 (m, 1H), 3.52–3.46 (m, 1H), 3.26–3.23 (m, 1H); ^13^C-NMR (100 MHz, CDCl_3_): δ_C_ 195.3, 177.2, 180.4, 139.0, 138.2, 135.8, 129.7, 129.5, 129.2, 128.8, 128.4, 128.5, 128.3, 128.0, 127.1, 126.6, 125.2, 122.6, 110.1, 83.0, 63.7, 61.5, 41.0. LC/MS (ESI) *m*/*z* 567 [M]^+^; Anal. Calcd for: C_32_H_23_Cl_2_N_3_O_3_: C, 67.61; H, 4.08; N, 7.39. Found: C, 67.74; H, 4.21; N, 7.43%.

*(2′R,3′R,4′S,5′S)-4′-Benzoyl-5′-benzyl-5-chlorospiro[3,2′]oxindolo-spiro[3,3″]oxindolopyrrolidine* (**5d**). Colorless solid (312 mg, 73%), mp 155–157 °C (EtOAc); *R*_F_ 0.4 (pet. ether/EtOAc, 1:1); IR (KBr): 1628, 1706 and 1710 cm^−1^; ^1^H-NMR (500 MHz, CDCl_3_): δ_H_ 9.79 (s, 1H, NH), 9.77 (s, 1H, NH), 7.47–6.30 (m, 17H, aromatic), 5.54–5.49 (m, 1H), 4.24 (d, *J* = 8.0, 1H), 3.18–3.02 (m, 2H); ^13^C-NMR (125 MHz, CDCl_3_): δ_C_ 196.8, 180.0, 177.0, 141.6, 141.2, 137.4, 138.6, 132.3, 129.5, 129.1, 129.0, 128.7, 128.4, 128.3, 128.0, 127.9, 127.1, 126.7, 126.1, 124.3, 123.7, 122.6, 121.7, 110.7, 110.0, 85.5, 64.9, 61.3, 59.8, 41.0. LC/MS (ESI) *m*/*z* 533 [M]^+^; Anal. Calcd for: C_32_H_24_ClN_3_O_3_: C, 71.97; H, 4.53; N, 7.87. Found: C, 71.89; H, 4.45; N, 7.95%.

*(2′S,3′S,4′S,5′S)-3′-Benzoyl-5′-benzyl-5-chlorospiro[3,2′]oxindolo-spiro[3″,4′]oxindolopyrrolidine* (**6d**). Colorless solid (21 mg, 5%), mp 144–145 °C (EtOAc); *R*_F_ 0.5 (pet. ether/EtOAc, 1:1); IR (KBr): 1628, 1706 and 1710 cm^−1^; ^1^H-NMR (500 MHz, CDCl_3_): δ_H_ 9.73 (s, 1H, NH), 9.16 (s, 1H, NH), 7.15–6.09 (m, 17H, aromatic), 5.22 (s, 1H), 4.76–4.72 (m, 1H), 3.32–3.30 (m, 1H), 3.03–2.99 (m, 1H); ^13^C-NMR (125 MHz, CDCl_3_): δ_C_ 197.7, 179.7, 174.5, 141.3, 141.1, 139.5, 137.2, 132.5, 131.9, 129.4, 129.2, 128.4, 128.2, 128.0, 127.5, 126.2, 126.0, 121.2, 110.3, 75.1, 64.8, 61.3, 39.6. LC/MS (ESI) *m*/*z* 533 [M]^+^; Anal. Calcd for: C_32_H_24_ClN_3_O_3_: C, 71.97; H, 4.53; N, 7.87. Found: C, 71.86; H, 4.49; N, 7.25%.

*(2′R,3′R,4′S,5′S)-4′-(4-Chlorobenzoyl)-5′-benzyl-5-nitrospiro[3,2′]oxindolo-spiro[3,3″]oxindolo-pyrrolidine* (**5e**). Reddish brown solid (294 mg, 72%), mp 232–234 °C (EtOAc); *R*_F_ 0.3 (pet. ether/EtOAc, 1:1); IR (KBr): 1625, 1708 and 1710 cm^−1^; ^1^H-NMR (400 MHz, CDCl_3_): δ_H_ 8.85 (s, 1H, NH), 8.62 (s, 1H, NH), 8.41–6.54 (m, 16H, aromatic), 5.54 (m, 1H),4.34 (d, *J* = 8.8 Hz, 1H), 3.16–3.05 (m, 2H); ^13^C-NMR (100 MHz, CDCl_3_): δ_C_ 195.2, 180.3, 177.5, 147.7, 143.3, 140.6, 139.3, 138.1, 135.6, 129.4, 129.3, 129.0, 128.7, 128.6, 126.7, 126.4, 123.6, 122.8, 122.0, 110.1, 110.0, 83.1, 63.7, 61.9, 59.4, 40.9. LC/MS (ESI) *m*/*z* 578 [M]^+^; Anal. Calcd for: C_32_H_23_ClN_4_O_5_: C, 66.38; H, 4.00; N, 9.68. Found: C, 66.47; H, 4.16; N, 9.75%.

*(2′S,3′S,4′S,5′S)-3′-(4-Chlorobenzoyl)-5′-benzyl-5-nitrospiro[3,2′]oxindolo-spiro[3″,4′]oxindolo-pyrrolidine* (**6e**). Reddish brown solid (20 mg, 5%), mp 210–212 °C (EtOAc); *R*_F_ 0.4 (pet. ether/EtOAc, 1:1); IR (KBr): 1625, 1708 and 1710 cm^−1^; ^1^H-NMR (400 MHz, CDCl_3_): δ_H_ 9.13 (s, 1H, NH), 7.99 (s, 1H, NH), 7.75–6.47 (m, 16H, aromatic), 5.31 (s, 1H), 5.03–5.01 (m, 1H), 3.51–3.45 (m, 1H), 3.28–3.25 (dd, 12.4, 4.4 Hz, 1H); ^13^C-NMR (100 MHz, CDCl_3_): δ_C_ 195.7, 180.8, 174.5, 147.5, 142.6, 139.5, 138.9, 135.3, 129.8, 129.4, 128.9, 128.9, 128.7, 128.5, 127.7, 128.0, 126.9, 125.4, 124.5, 123.2, 110.2, 75.1, 65.0, 61.0, 42.1. LC/MS (ESI) *m*/*z* 578 [M]^+^; Anal. Calcd for: C_32_H_23_ClN_4_O_5_: C, 66.38; H, 4.00; N, 9.68. Found: C, 66.49; H, 4.14; N, 9.79%.

*(2′R,3′R,4′S,5′S)-4′-Benzoyl-5′-benzyl-5-nitrospiro[3,2′]oxindolo-spiro[3,3″]oxindolopyrrolidine* (**5f**). Brown solid (310 mg, 71%), mp 197–199 °C (EtOAc); *R*_F_ 0.3 (pet. ether/EtOAc, 1:1); IR (KBr): 1625, 1708 and 1710 cm^−1^; ^1^H-NMR (500 MHz, CDCl_3_): δ_H_ 8.33 (s, 1H, NH) 7.96 (s, 1H, NH), 7.65–6.31 (m, 17H, aromatic), 5.30–5.25 (m, 1H), 4.32 (d, *J* = 8.7 Hz, 1H), 3.06–3.02 (m, 2H); ^13^C-NMR (125 MHz, CDCl_3_): δ_C_ 195.5, 180.5, 176.2, 149.3, 140.8, 141.7, 137.7, 136.4, 132.8, 129.6, 129.4, 128.8, 128.5, 128.4, 128.0, 126.4, 125.6, 125.2, 123.1, 120.8, 109.4, 83.1, 68.1, 61.2, 55.9, 40.1. LC/MS (ESI): *m*/*z* 544 [M]^+^; Anal. Calcd for: C_32_H_24_N_4_O_5_: C, 70.58; H, 4.44; N, 10.29. Found: C, 70.66; H, 4.37; N, 10.18%.

*(2′S,3′S,4′S,5′S)-3′-Benzoyl-5′-benzyl-5-nitrospiro[3,2′]oxindolo-spiro[3″,4′]oxindolopyrrolidine* (**6f**). Brown needles (21 mg, 5%), mp 175–177 °C (EtOAc/DMSO, 9:1); *R*_F_ 0.4 (pet. ether/EtOAc, 1:1); IR (KBr): 1625, 1708 and 1710 cm^−1^; ^1^H-NMR (500 MHz, CDCl_3_): δ_H_ 10.28 (s, 1H, NH), 9.24 (s, 1H, NH), 7.70–6.22 (m, 17H, aromatic), 4.76–4.74 (m, 1H), 5.15 (s, 1H), 3.28–3.25 (m, 1H), 2.93–2.88 (m, 1H); ^13^C-NMR (125 MHz, CDCl_3_): δ_C_ 197.3, 180.3, 174.1, 148.9, 141.1, 137.3, 132.7, 130.8, 129.4, 128.4, 128.2, 127.9, 127.5, 126.2, 126.0, 124.9, 121.5, 110.0, 109.3, 74.7, 64.9, 61.2, 55.8, 39.9. LC/MS (ESI) *m*/*z* 544 [M]^+^; Anal. Calcd for: C_32_H_24_N_4_O_5_: C, 70.58; H, 4.44; N, 10.29. Found: C, 70.68; H, 4.35; N, 10.21%.

### 3.3. X-ray Crystallography

A clear intense yellow block-like specimen of C_37_H_36_N_3_O_7_S_2_, approximate dimensions 0.39 × 0.457 × 0.674 mm, was used for the X-ray crystallographic analysis. A total of 684 frames were collected. The total exposure time was 2.85 h. The frames were integrated with the Bruker SAINT software package [35] using a narrow-frame algorithm. The integration of the data using a monoclinic unit cell, space group P2_1_/n, yielded a total of 35419 reflections to a maximum θ angle of 28.33° (0.75 Å resolution), of which 8504 were independent (average redundancy 5.743, completeness = 99.6%, R_int_ = 3.18%, R_sig_ = 2.93%) and 6264 (76.53%) were greater than 2σ(*F^2^*). The final cell constants of *a* = 13.5273(5) Å, *b* = 14.8582(6) Å, *c* = 17.1768(7) Å, β = 95.0630(10)°, volume = 3437.3(2) Å^3^, are based upon the refinement of the XYZ-centroids of 9790 reflections above 20 σ(I) with 4.588° < 2θ < 56.36°. Data were corrected for absorption effects using the multi-scan method (SADABS). The ratio of minimum to maximum apparent transmission was 0.907. The calculated minimum and maximum transmission coefficients (based on crystal size) are 0.9490 and 0.9690.

The final anisotropic full-matrix least-squares refinement on *F^2^* with 388 variables converged at R_1_ = 6.41%, for the observed data and wR_2_ = 20.09% for all data. The goodness-of-fit was 1.06. The largest peak in the final difference electron density synthesis was 8.210 e-/Å^3^ and the largest hole was −1.079 e-/Å^3^ with an RMS deviation of 0.325 e-/Å^3^. On the basis of the final model, the calculated density was 1.067 g/cm^3^ and *F*(000) 1468.

## 4. Conclusions

A one-pot three component 1,3-dipolar cycloaddition reaction of azomethine ylide generated *in situ* from substituted isatins and L-phenylalanine to (*E*)-2-oxoindolino-3-ylidene acetophenones in [bmim]BF_4_ afforded the dispirooxindolopyrrolidines **5a**–**f** and **6a**–**f** in good yields with high selectivity, the former isomer being obtained predominantly. This methodology gains importance as the reaction was completed in shorter reaction times with better yields/selectivity besides the recyclability of the green solvent [bmim]BF_4_.

## Supplementary Materials

Supplementary materials can be accessed at: http://www.mdpi.com/1420-3049/20/01/0780/s1.

## References

[1] Welton T. (1999). Room-temperature ionic liquids. Solvents for synthesis and catalysis. Chem. Rev..

[2] Petkovic M., Seddon K.R., Rebelo L.P.N., Pereira C.S. (2011). Ionic liquids: A pathway to environmental acceptability. Chem. Soc. Rev..

[3] Yao Q. (2002). OsO_4_ in ionic liquid [Bmim]PF_6_: A recyclable and reusable catalyst system for olefin dihydroxylation. Remarkable effect of DMAP. Org. Lett..

[4] Kumar A., Pawar S.S. (2004). Converting *exo*-selective Diels-Alder reaction to *endo*-selective in chloroloaluminate ionic liquids. J. Org. Chem..

[5] Fukumoto K., Yoshizawa M., Ohno H. (2005). Room temperature ionic liquids from 20 natural amino acids. J. Am. Chem. Soc..

[6] Forsyth S.A., Frohlich U., Goodrich P., Gunaratne H.Q.N., Hardacre C., McKeown A., Seddon K.R. (2010). Functionalised ionic liquids: Synthesis of ionic liquids with tethered basic groups and their use in Heck and Knoevenagel reactions. New J. Chem..

[7] Gao J., Song Q.-W., He L.-N., Liu C., Yang Z.-Z., Han X., Li X.-D., Song Q.-C. (2012). Preparation of polystyrene-supported Lewis acidic Fe(III) ionic liquid and its application in catalytic conversion of carbon dioxide. Tetrahedron.

[8] Narayana Kumar G.G.K.S., Aridoss G., Laali K.K. (2012). Condensation of propargylic alcohols with indoles and carbazole in [bmim][PF_6_]/Bi(NO_3_)_3_·5H_2_O: A simple high yielding propargylation method with recycling and reuse of the ionic liquid. Tetrahedron Lett..

[9] Ramon D.J., Yus M. (2005). Asymmetric multicomponent reactions (AMCRs): The new frontier. Angew. Chem. Int. Ed..

[10] Sunderhaus J.D., Martin S.F. (2009). Applications of multicomponent reactions to the synthesis of diverse heterocyclic scaffolds. Chem. Eur. J..

[11] Isambert N., Lavilla R. (2008). Heterocycles as key substrates in multicomponent reactions: The fast lane towards molecular complexity. Chem. Eur. J..

[12] Orru R.V.A., de Greef M. (2003). Recent advances in solution-phase multicomponent methodology for the synthesis of heterocyclic compounds. Synthesis.

[13] Isambert N., Duque M.M.S., Plaquevent J.-C., Genisson Y., Rodriguez J., Constantieux T. (2011). For a review of MCRs in ionic liquids, multicomponent reactions and ionic liquids: A perfect synergy for eco-compatible heterocyclic synthesis. Chem. Soc. Rev..

[14] Okita T., Isobe M. (1994). Synthesis of the pentacyclic intermediate for dynemicin A and unusual formation of spiro-oxindole ring. Tetrahedron.

[15] Kornet M.J., Thio A.P. (1976). Oxindole-3-spiropyrrolidines and -piperidines. Synthesis and local anesthetic activity. J. Med. Chem..

[16] Skiles J.W., McNeil D. (1990). Spiro indolinone beta-lactams, inhibitors of poliovirus and rhinovirus 3C-proteinases. Tetrahedron Lett..

[17] Jossang A., Jossang P., Hadi H.A., Sevenet T., Bodo B. (1991). Horsfiline, an oxindole alkaloid from Horsfieldia superba. J. Org. Chem..

[18] Galliford C.V., Scheidt K.A. (2007). Pyrrolidinyl-spirooxindole natural products as inspirations for the development of potential therapeutic agents. Angew. Chem. Int. Ed. Engl..

[19] James M.N.G., Williams G.J.B. (1972). The molecular and crystal structure of an oxindole alkaloid (6-hydroxy-2'-(2-methylpropyl)-3,3'-spirotetrahydropyrrolidino-oxindole). Can. J. Chem..

[20] Cui C.-B., Kakeya H., Okada G., Onose R., Osada H. (1996). Novel mammalian cell cycle inhibitors, tryprostatins A, B and other diketopiperazines produced *Aspergillus fumigatus*. I. Taxonomy, fermentation, isolation and biological properties. J. Antibiot..

[21] Cui C.-B., Kakeya H., Osada H. (1996). Novel mammalian cell cycle inhibitors, spirotryprostatins A and B, produced by *Aspergillus fumigatus*, which inhibit mammalian cell cycle at G2/M phase. Tetrahedron.

[22] Khafagy M.M., Abd El-Wahab A.H.F., Eid F.A., El-Agrody A.M. (2002). Synthesis of halogen derivatives of benzo[*h*]chromene and benzo[*a*]anthracene with promising antimicrobial activities. Farmaco.

[23] Kang T.H., Matsumoto K., Tohda M., Murakami Y., Takayama H., Kitajima M., Aimi N., Watanabe H. (2002). Pteropodine and isopteropodine positively modulate the function of rat muscarinic M_1_ and 5-HT_2_ receptors expressed in *Xenopus* oocyte. Eur. J. Pharmacol..

[24] García Prado E., García Gimenez M.D., de la Puerta Vázquez R., Espartero Sánchez J.L., Sáenz Rodríguez M.T. (2007). Antiproliferative effects of mitraphylline, a pentacyclic oxindole alkaloid of *Uncaria tomentosa* on human glioma and neuroblastoma cell lines. Phytomedicine.

[25] Arumugam N., Raghunathan R. (2011). Facile synthesis of β-lactam-grafted spirooxindolopyrrolidine through regioselective 1,3-dipolar cycloaddition reaction. Synth. Commun..

[26] Arumugam N., Jayashankaran J., Rathna Durga R.S.M., Raghunathan R. (2005). A novel access to highly functionalised β-lactams by regio- and stereoselective 1,3-dipolar cycloaddition reaction. Tetrahedron.

[27] Arumugam N., Raghunathan R., Shanmugaiah V., Mathivanan N. (2010). Synthesis of novel β-lactam fused spiroisoxazolidine chromanones and tetralones as potent antimicrobial agent for human and plant pathogens. Bioorg. Med. Chem. Lett..

[28] Arumugam N., Periyasami G., Raghunathan R., Kamalraj S., Muthumary J. (2011). Synthesis and antimicrobial activity of highly functionalised novel β-lactam grafted spiropyrrolidines and pyrrolizidines. Eur. J. Med. Chem..

[29] Arumugam N., Raghunathan R., Almansour A.I., Karama U. (2012). An efficient synthesis of highly functionalized novel chromeno[4,3-*b*]pyrroles and indolizino[6,7-*b*]indoles as potent antimicrobial and antioxidant agents. Bioorg. Med. Chem. Lett..

[30] Arumugam N., Almansour A.I., Suresh Kumar R., Perumal S., Ghabbour H.A., Fun H.-K. (2013). A 1,3-dipolar cycloaddition–annulation protocol for the expedient regio-, stereo- and product-selective construction of novel hybrid heterocycles comprising seven rings and seven contiguous stereocentres. Tetrahedron Lett..

[31] Suresh Babu A.R., Raghunathan R. (2007). ZrOCl·8H_2_O mediated microwave induced [3+2] cycloaddition of azomethine ylides-a facile one-pot synthesis of novel dispiroheterocycles. Tetrahedron Lett..

[32] Suresh Babu A.R., Raghunathan R. (2007). Ultrasonic assisted-silica mediated [3+2] cycloaddition of azomethine ylides-a facile multicomponent one-pot synthesis of novel dispiroheterocycles. Tetrahedron Lett..

[33] Suresh Babu A.R., Raghunathan R. (2008). An easy access to novel steroidal dispiropyrrolidines through 1,3-dipolar cycloaddition of azomethine ylides. Tetrahedron Lett..

[B34-molecules-20-00780] 34.Crystallographic data (excluding structure factors) for dispiro compound **6f** in this article have been deposited with the Cambridge Crystallographic Data Centre as supplementary publication number CCDC 1026186–1026187. Copies of the data can be obtained, free of charge, on application to CCDC, 12 Union Road, Cambridge CB2 1EZ, UK [fax: +44 (0)1223 336033 or e-mail: deposit@ccdc.cam.ac.uk].

[35] Sheldrick G.M. (2008). A short history of SHELX. Acta Crystallogr. Sect. A: Found. Crystallogr..

