# Supplementary material for: A Novel One-Pot Green Synthesis of Dispirooxindolo-pyrrolidines via1,3-Dipolar Cycloaddition Reactions of Azomethine Ylides
Source: Molecules. 2015 Jan 7;20(1):780–91. doi: 10.3390/molecules20010780 (PMC6272743; doi:10.3390/molecules20010780)
Supplement: Supplementary file 1 [file molecules-20-00780-s001.pdf]

Supplementary Materials

Table S1. Selected geometric parameters (Å, °).

**S1A—C37A**	1.748 (6)	O2—C27	1.220 (3)
**S1A—O7A**	1.518 (3)	O3—C26	1.225 (3)
**S1A—C36A**	1.769 (3)	O4—N3	1.222 (3)
**S2—O6**	1.471 (2)	O5—N3	1.216 (3)
**S2—C35**	1.735 (5)	N1—C8	1.366 (3)
**S2—C34**	1.727 (6)	N1—C1	1.385 (3)
**S1B—C36B**	1.75 (3)	N2—C7	1.473 (3)
**S1B—C37B**	1.68 (3)	N2—C11	1.459 (3)
**S1B—O7B**	1.49 (3)	N3—C4	1.456 (3)
**O1—C8**	1.216 (3)		
**C36A—S1A—C37A**	99.2 (2)	N1—C1—C2	128.3 (2)
**O7A—S1A—C37A**	104.8 (2)	N3—C4—C5	118.7 (2)
**O7A—S1A—C36A**	107.06 (17)	N3—C4—C3	118.1 (2)
**O6—S2—C35**	110.2 (2)	N2—C7—C8	107.08 (17)
**C34—S2—C35**	97.3 (3)	N2—C7—C6	112.92 (17)
**O6—S2—C34**	107.1 (3)	N2—C7—C9	103.96 (16)
**C36B—S1B—C37B**	108.4 (19)	N1—C8—C7	107.15 (18)
**O7B—S1B—C36B**	110.9 (17)	O1—C8—N1	125.5 (2)
**O7B—S1B—C37B**	117 (2)	O1—C8—C7	127.2 (2)
**C1—N1—C8**	112.46 (18)	N2—C11—C12	112.29 (18)
**C7—N2—C11**	105.40 (17)	N2—C11—C10	107.19 (16)
**O4—N3—C4**	118.7 (2)	O3—C26—C10	125.82 (19)
**O4—N3—O5**	122.7 (2)	O3—C26—C25	126.1 (2)
**O5—N3—C4**	118.5 (2)	O2—C27—C28	120.8 (2)
**N1—C1—C6**	109.51 (19)	O2—C27—C9	121.1 (2)

Table S2. Hydrogen-bond geometry (Å, °).

D—H···*A*	*D*—H	H···*A*	*D*···*A*	*D*—H···*A*
**N1—H1N1···O7A** ^{i}	0.84 (3)	1.97 (3)	2.779 (3)	162 (3)
**N2—H1N2···O3** ^{ii}	0.86 (3)	2.26 (3)	3.120 (2)	176 (2)
**C3—H3A···O2** ^{iii}	0.9300	2.6000	3.235 (3)	126.00
**C9—H9A···O3**	0.9800	2.4800	2.992 (3)	112.00
**C11—H11A···O1**	0.9800	2.5000	3.083 (3)	118.00
**C12—H12B···O5** ^{ii}	0.9700	2.4800	3.247 (3)	136.00
**C20—H20A···O1**	0.9300	2.4300	3.241 (3)	145.00
**C25—H25A···O6** ^{iv}	0.9300	1.9000	2.800 (3)	161.00
**C31—H31A···O7A** ^{v}	0.9300	2.4900	3.364 (4)	156.00
**C37A—H37C···O4** ^{vi}	0.9600	2.4300	3.202 (6)	138.00

Symmetry codes: (i) *x*+1, *y*, *z*; (ii) −*x*+1, −*y*+1, −*z*; (iii) −*x*+2, −*y*+1, −*z*; (iv) *x*, *y*, *z*−1; (v) *x*+1/2, −*y*+1/2, *z*−1/2; (vi) *x*−1/2, −*y*+3/2, *z*+1/2.

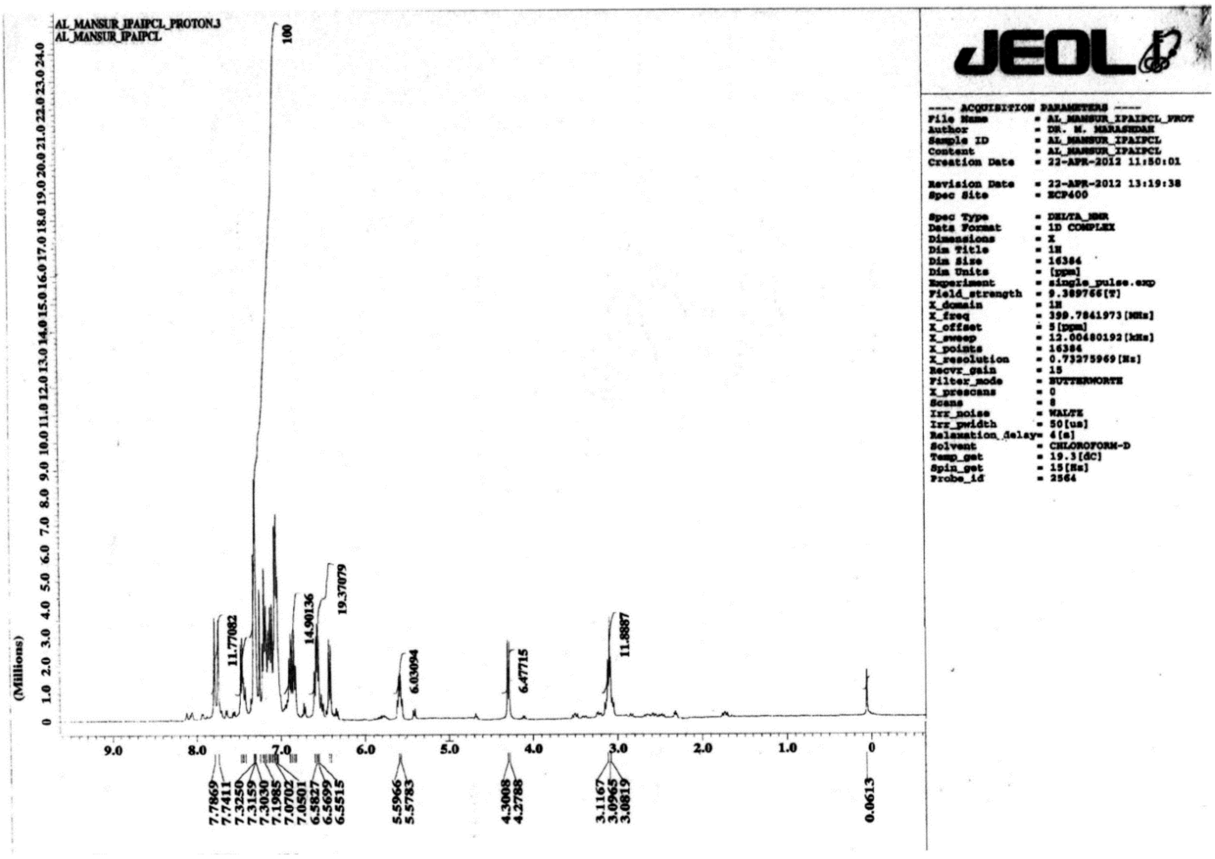

Figure S1. ^{1}H-NMR spectrum of **5a**.

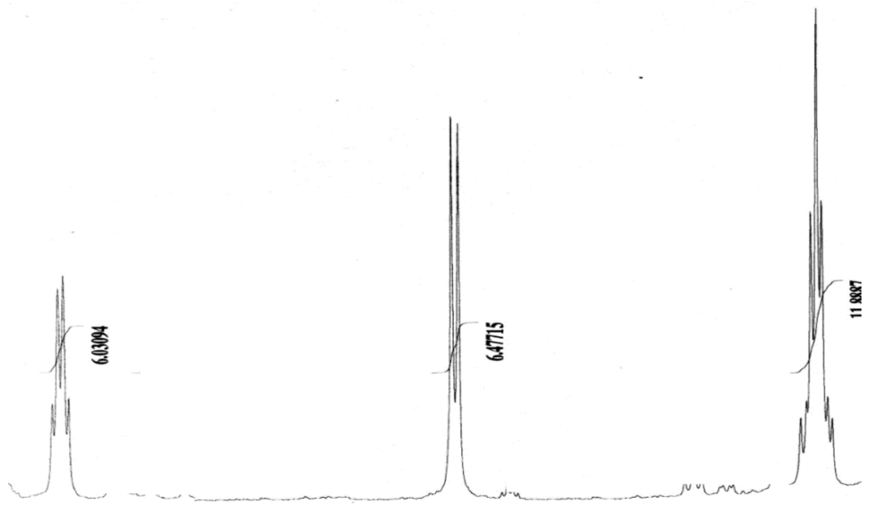

Figure S2. Expanded ^{1}H-NMR spectrum of **5a**.

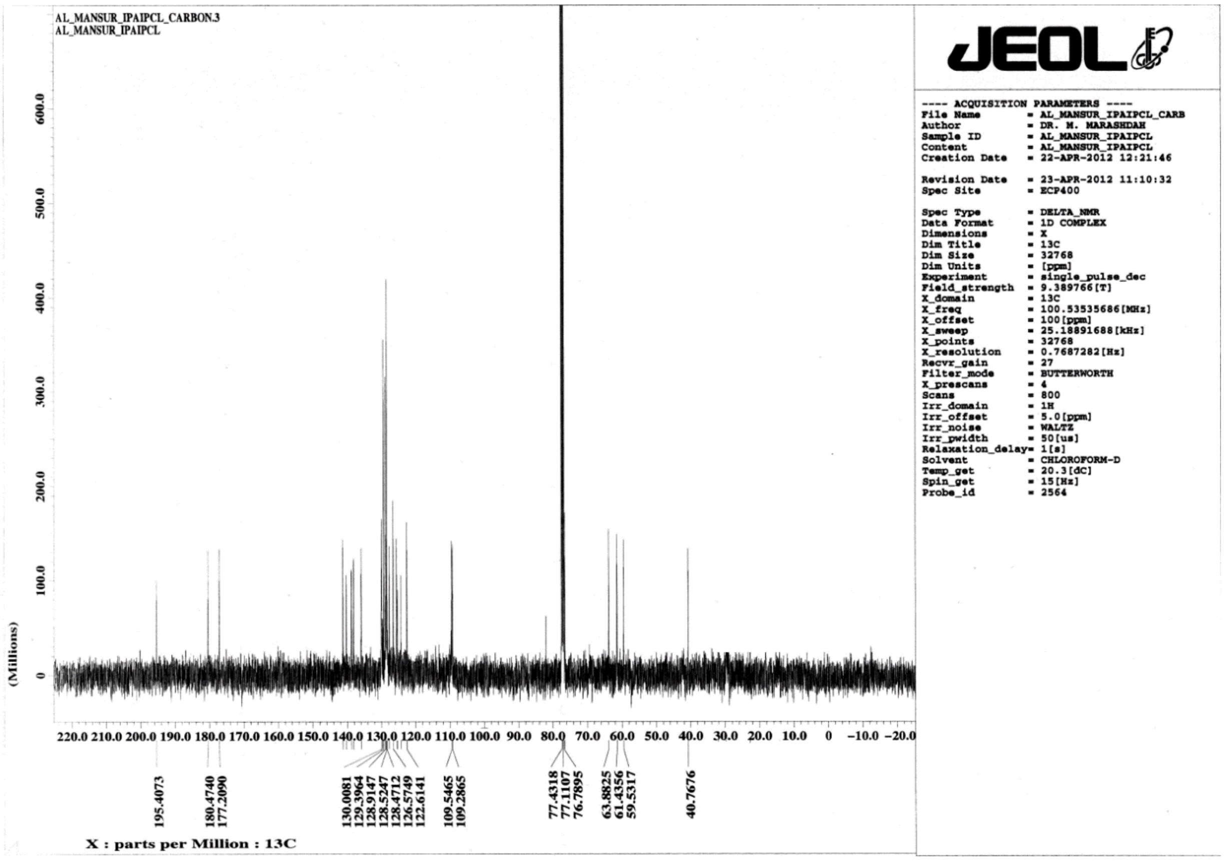

Figure S3. ^{13}C-NMR spectrum of **5a**.

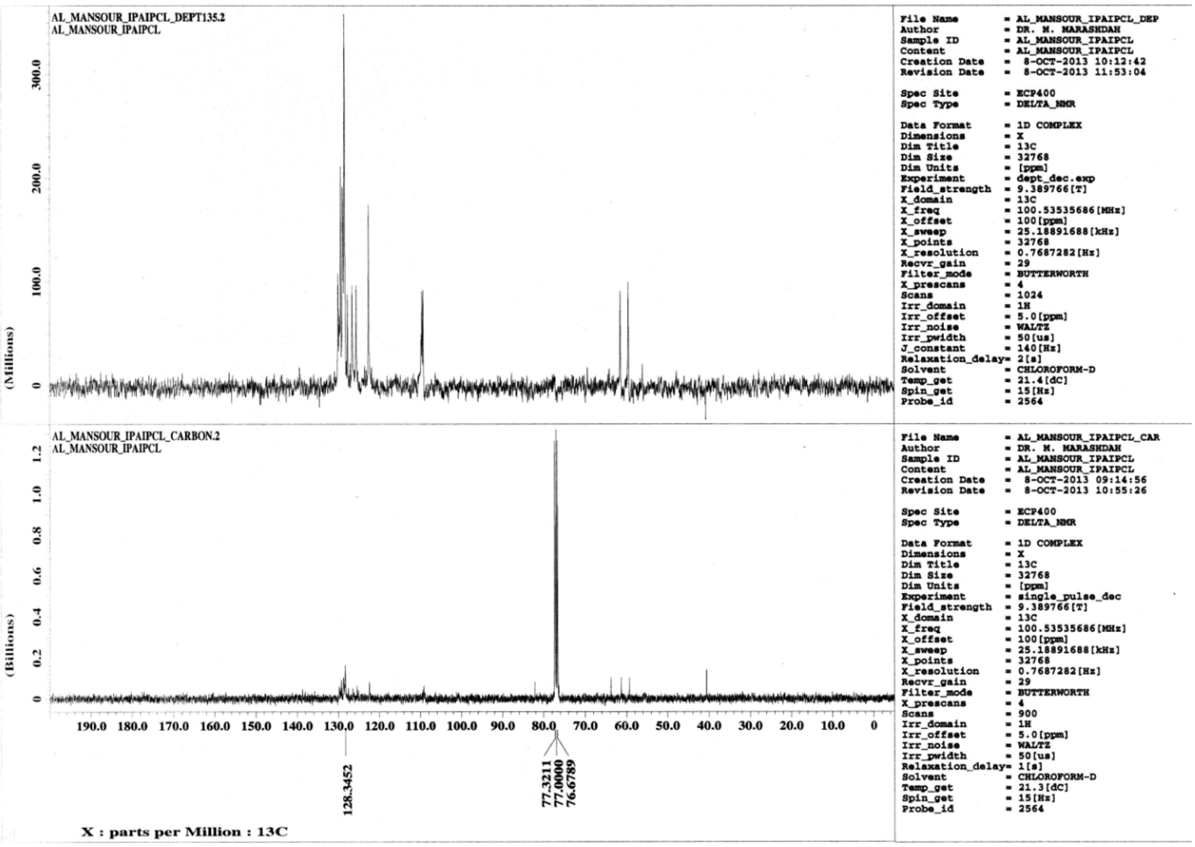

Figure S4. DEPT-135 spectrum of **5a**.

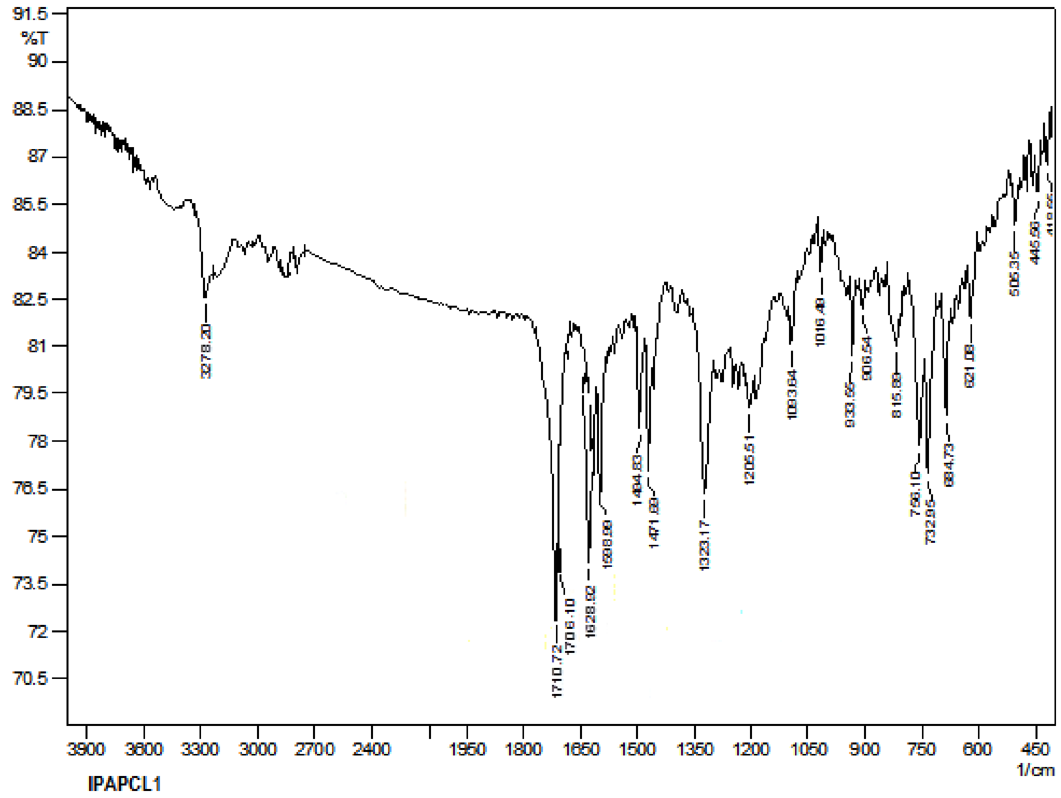

Figure S5. IR spectrum of **5a**.

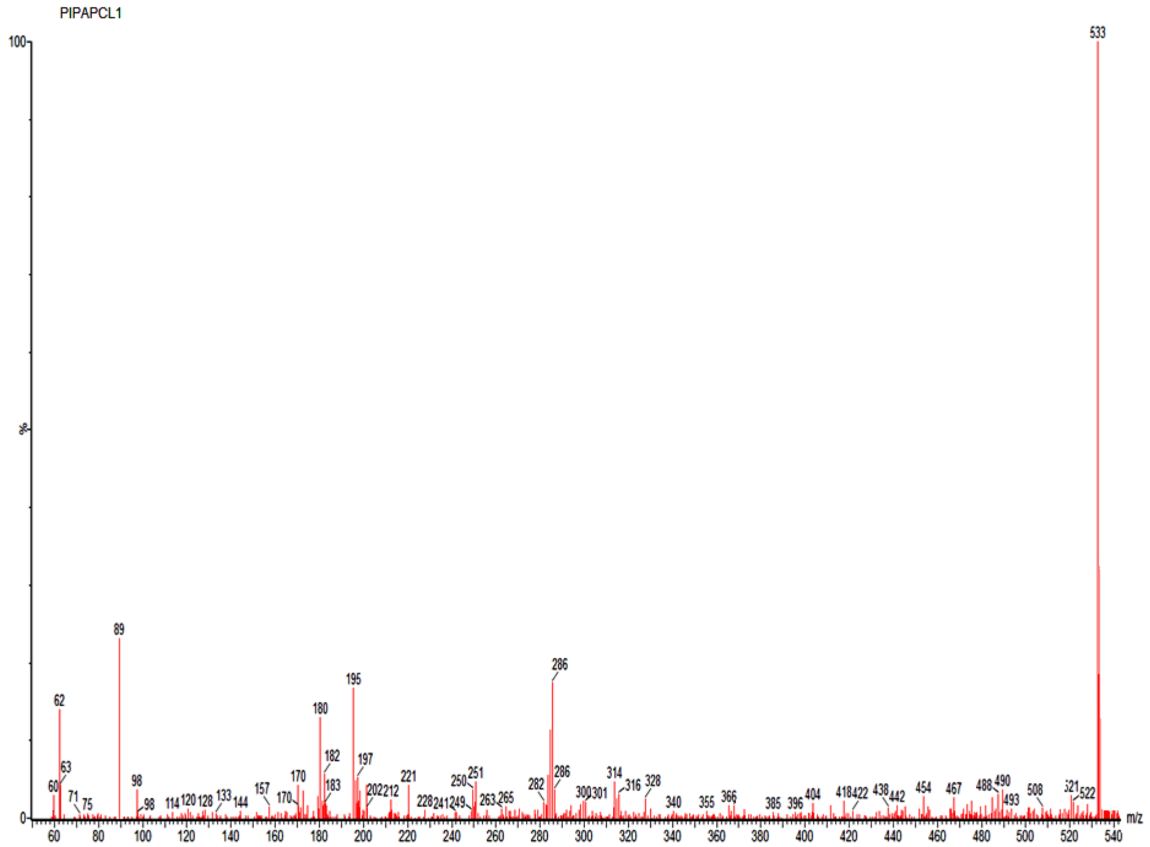

Figure S6. Mass spectrum of **5a**.

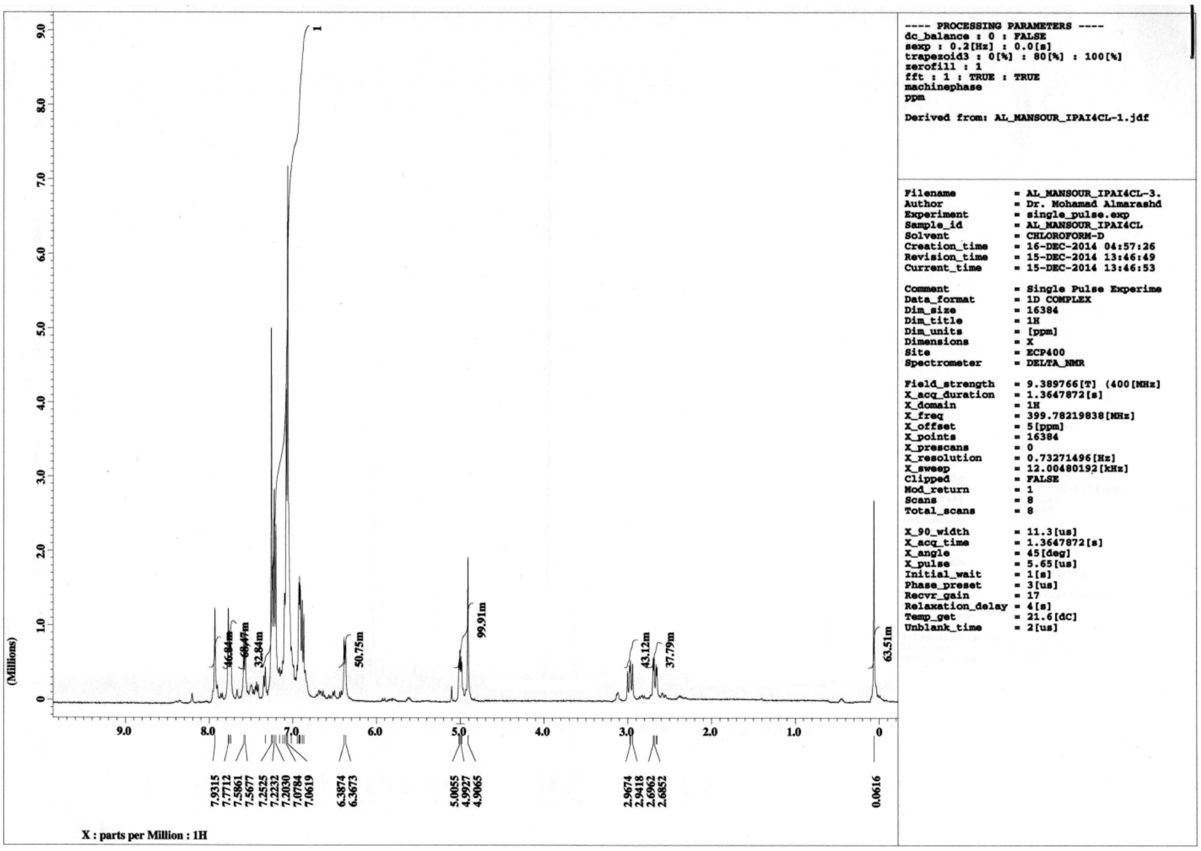

Figure S7. ^{1}H-NMR spectrum of **6a**.

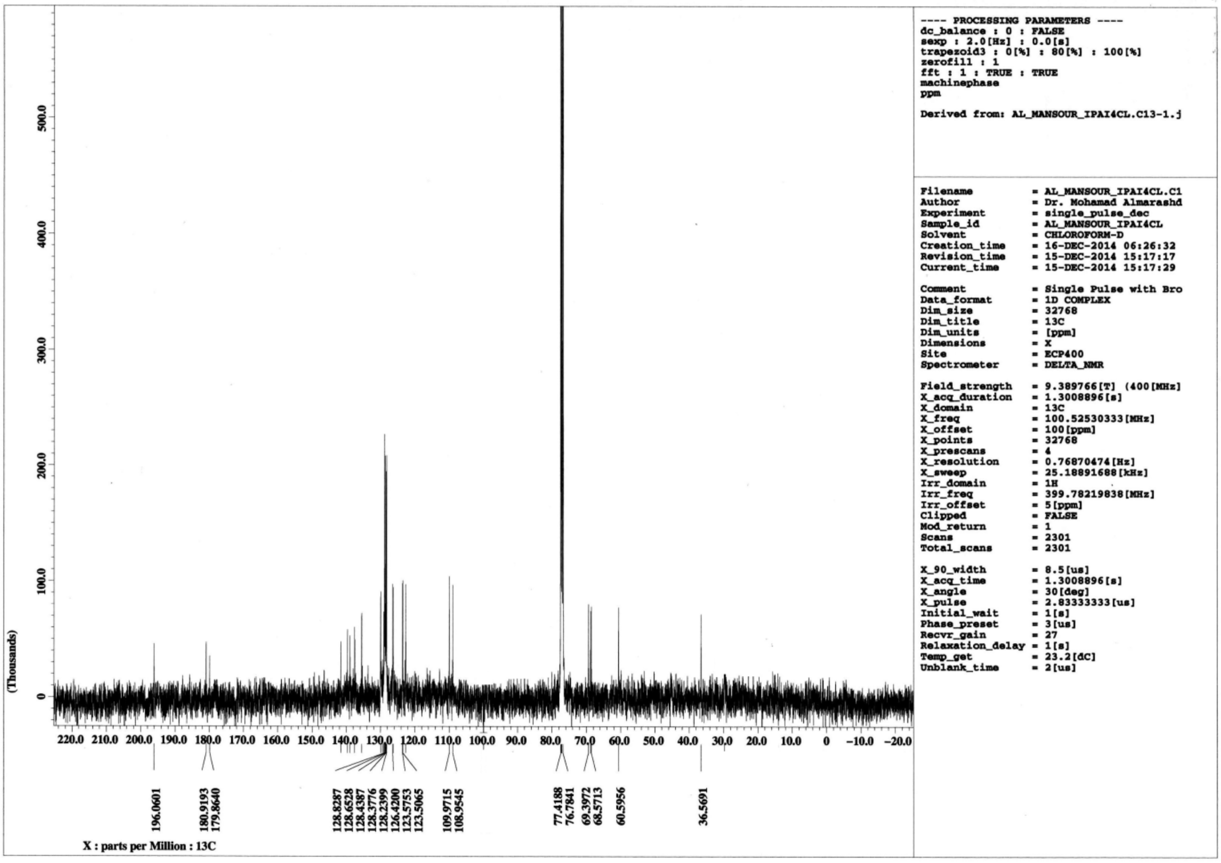

Figure S8. ^{13}C-NMR spectrum of **6a**.

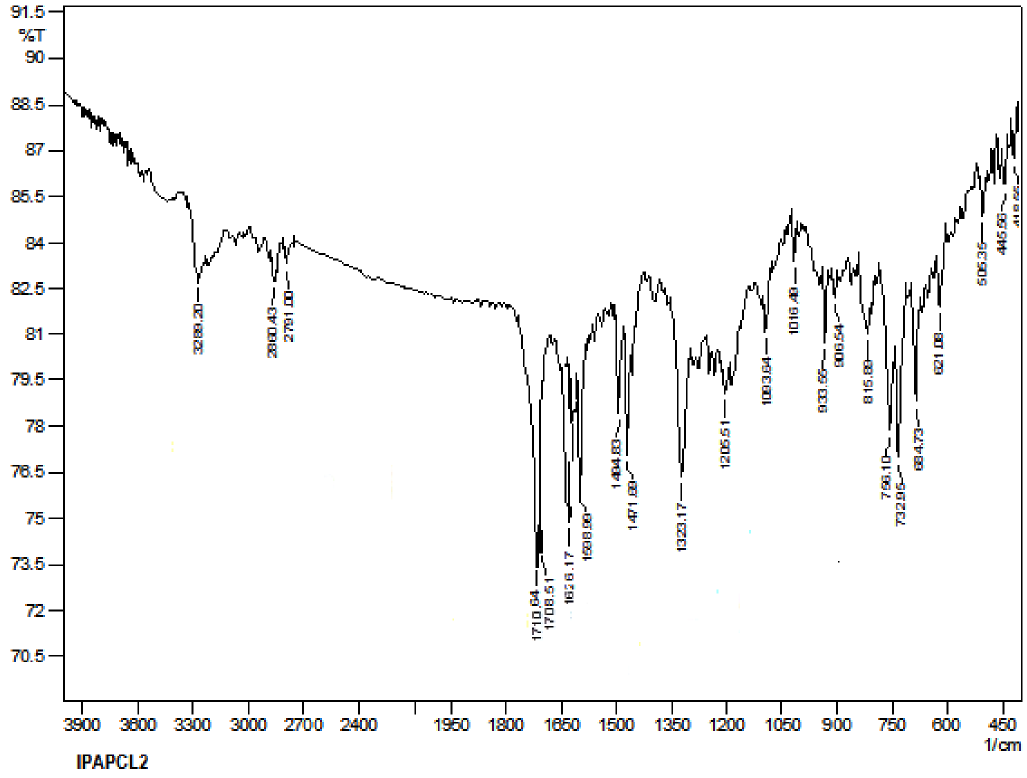

Figure S9. IR spectrum of **6a**.

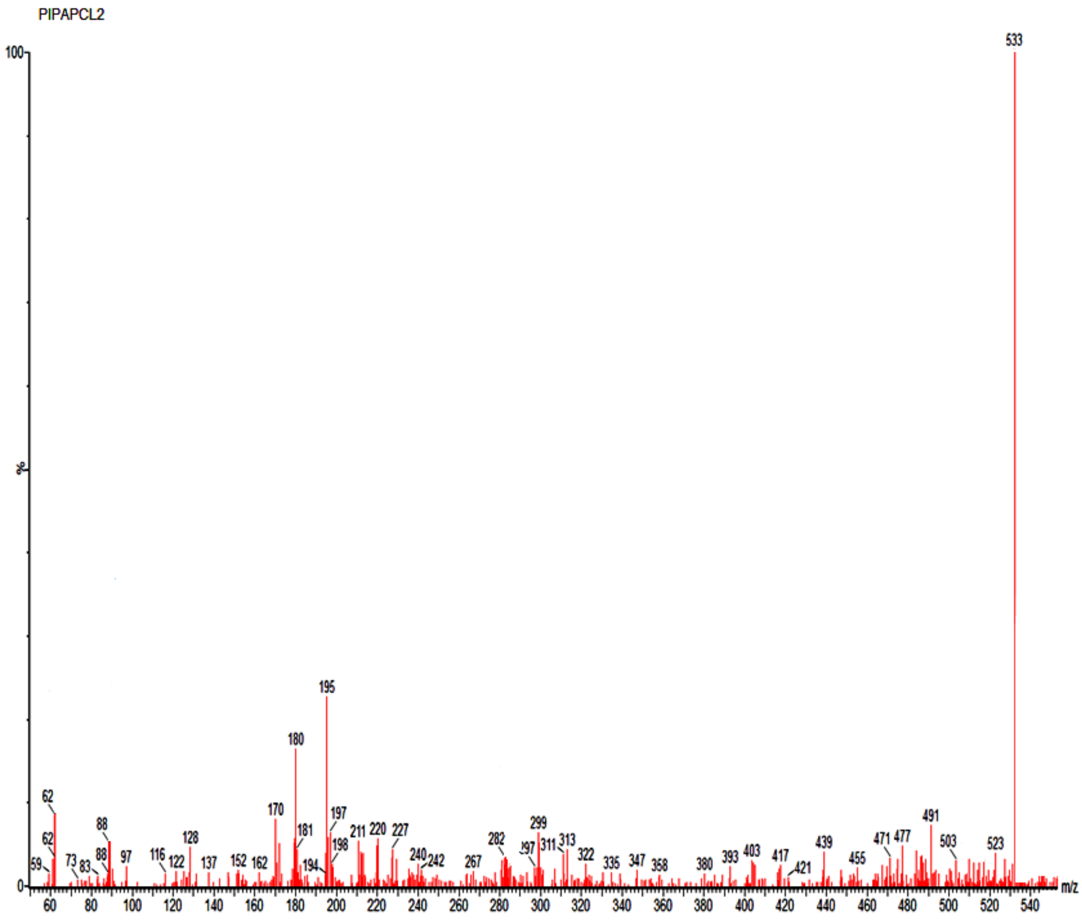

Figure S10. Mass spectrum of **6a**.

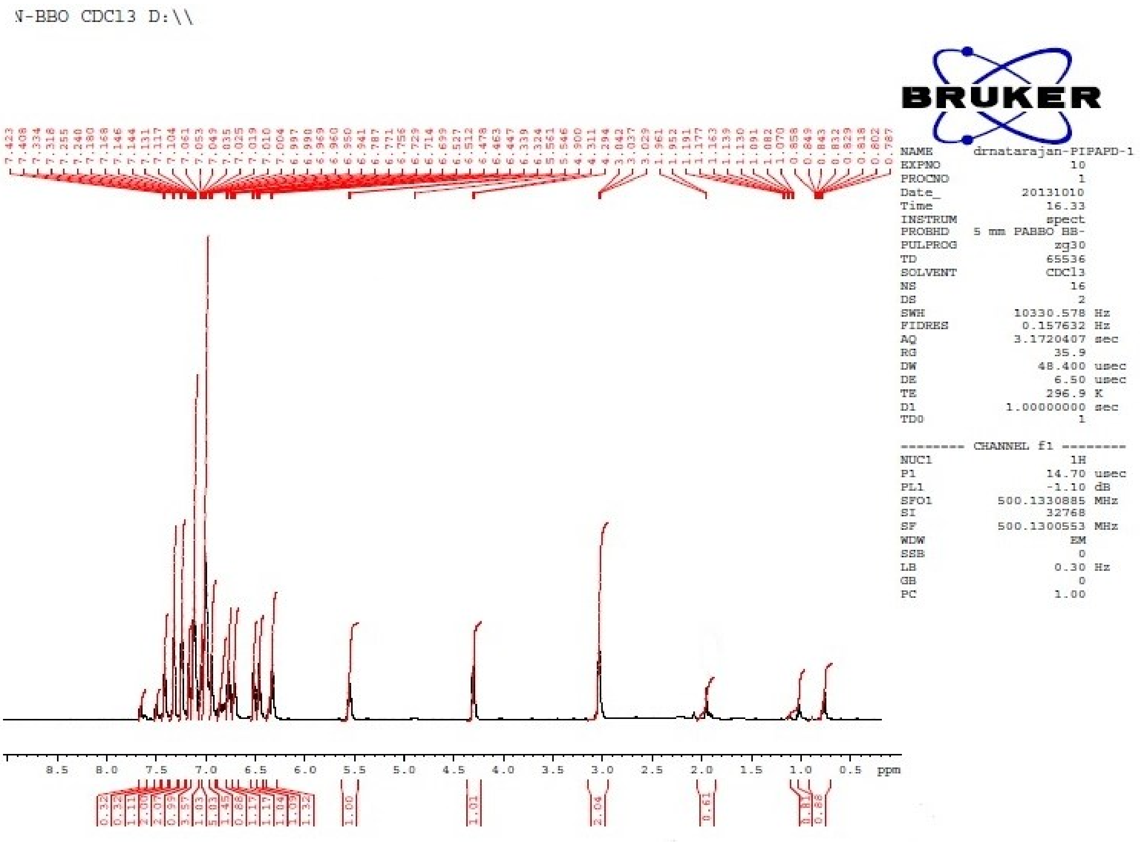

Figure S11. ^{1}H-NMR spectrum of **5b**.

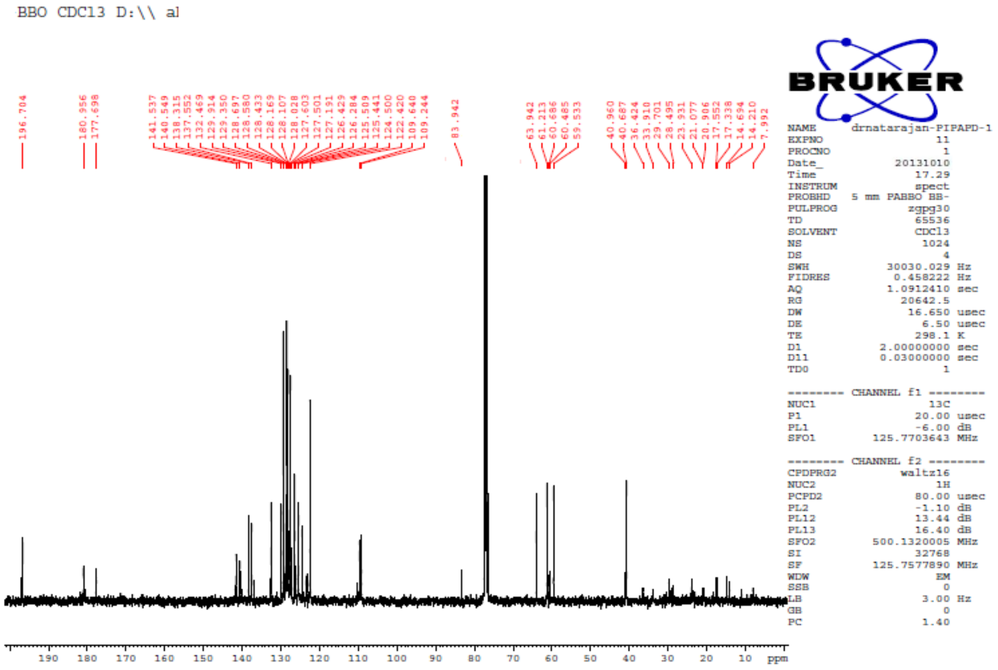

Figure S12. ^{13}C-NMR spectrum of **5b**.

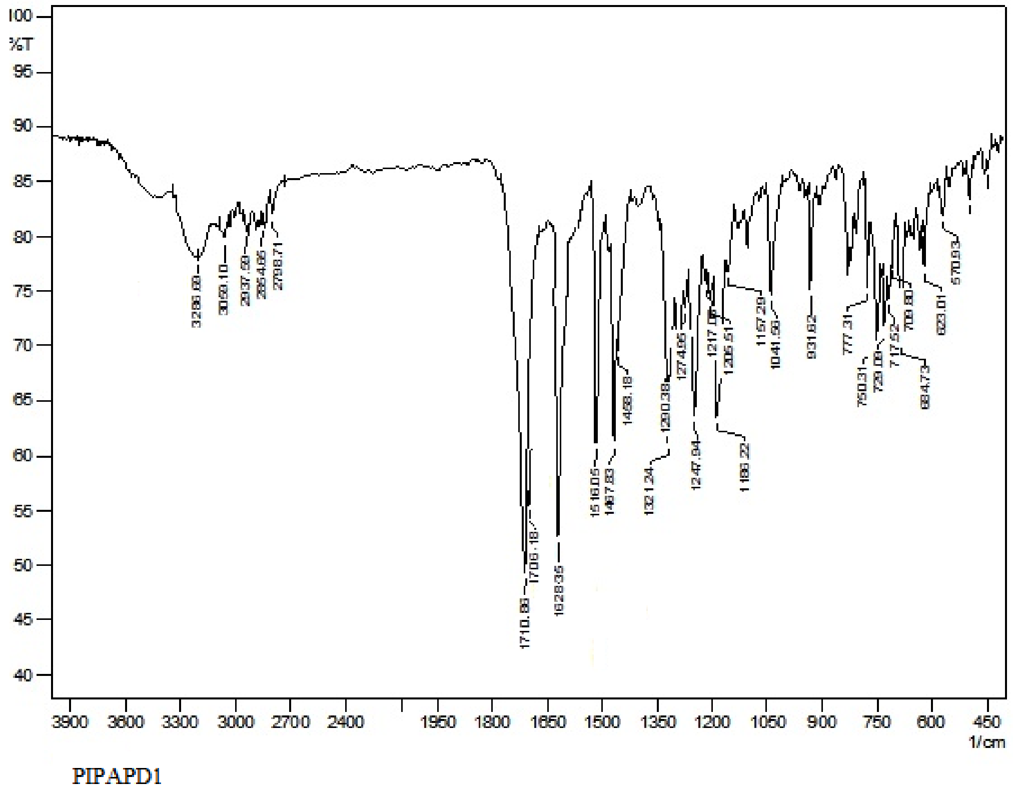

Figure S13. IR spectrum of **5b**.

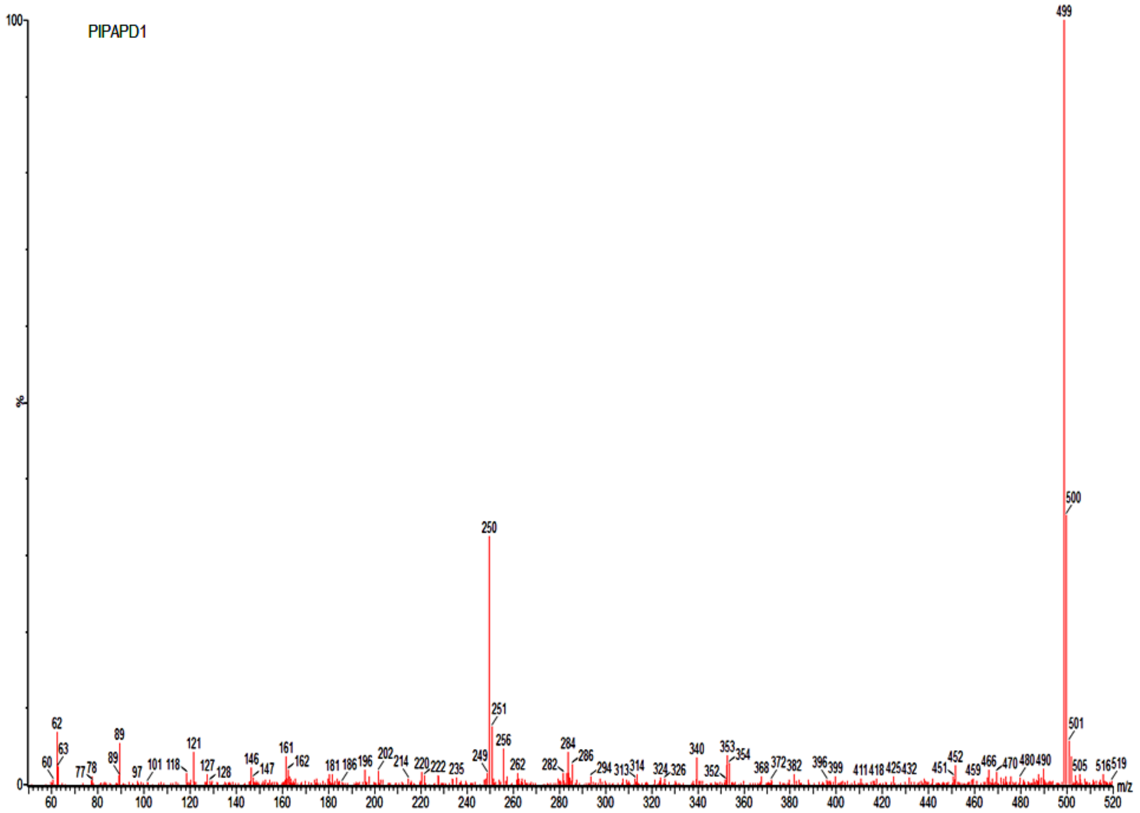

Figure S14. Mass spectrum of **5b**.

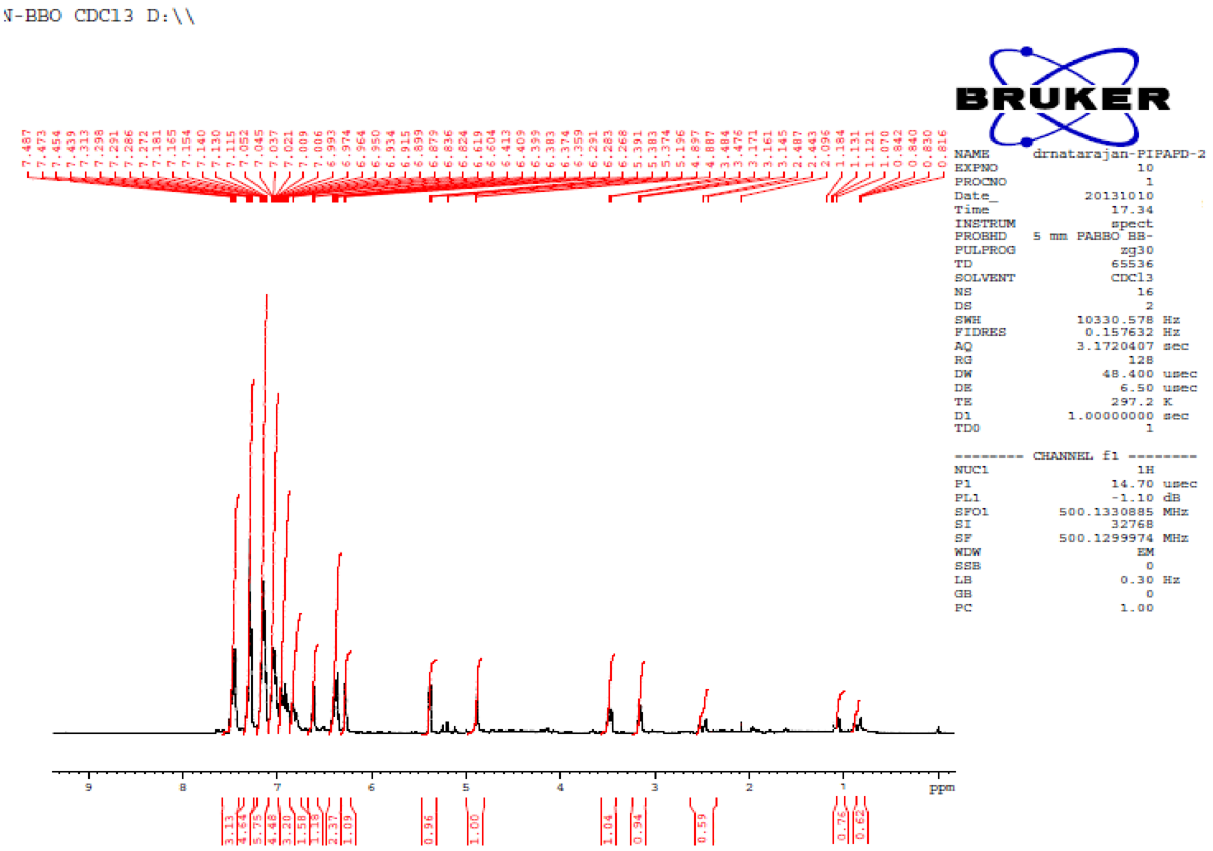

Figure S15. ^{1}H-NMR spectrum of **6b**.

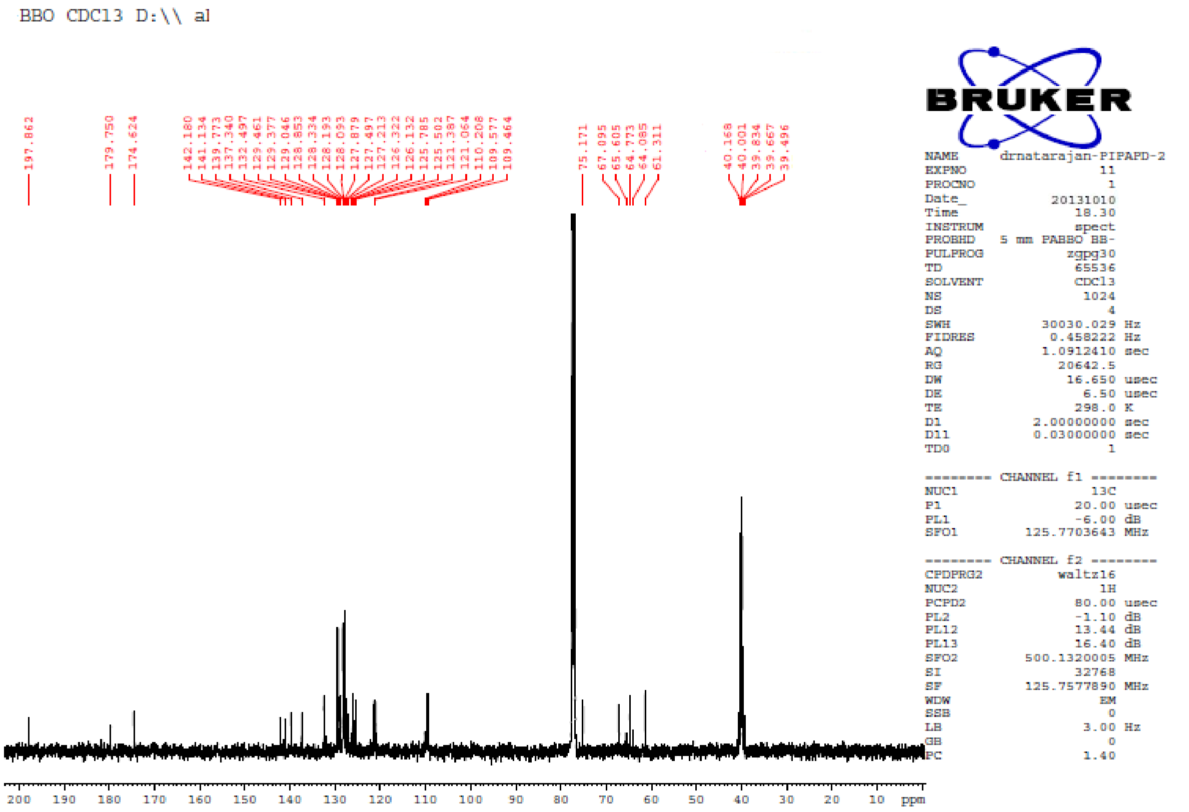

Figure S16. ^{13}C-NMR spectrum of **6b**.

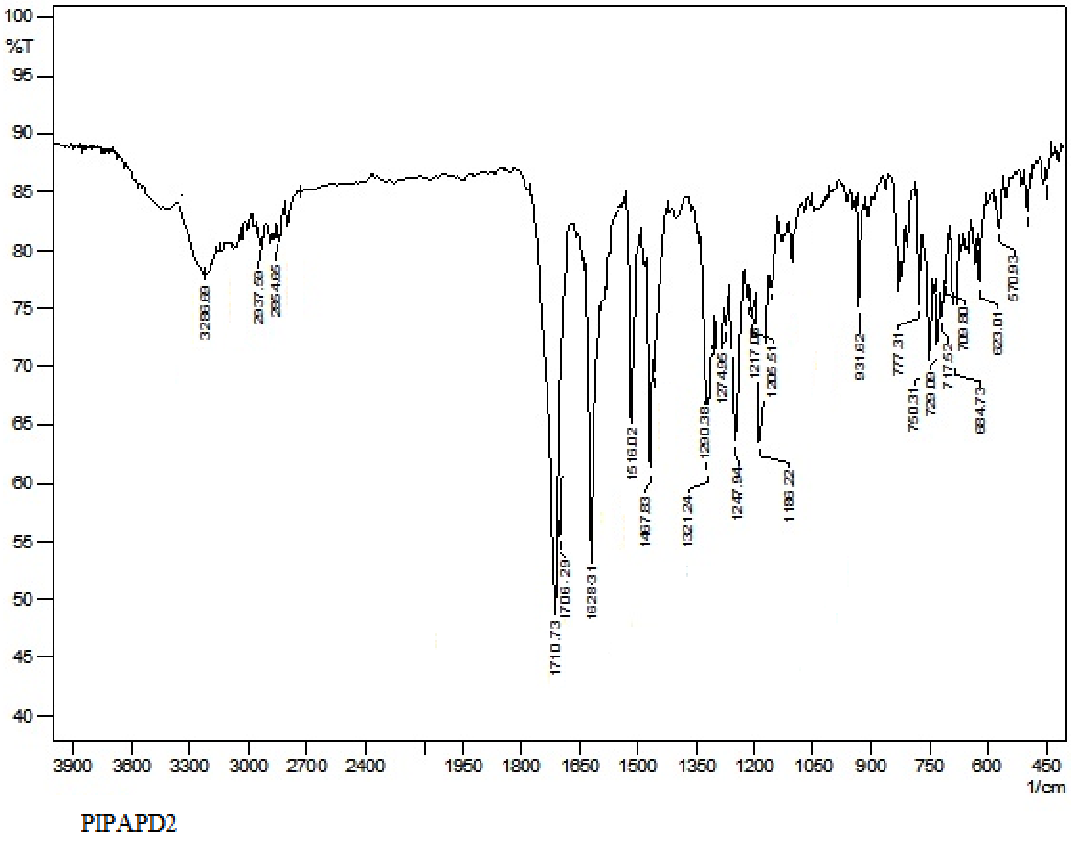

Figure S17. IR spectrum of **6b**.

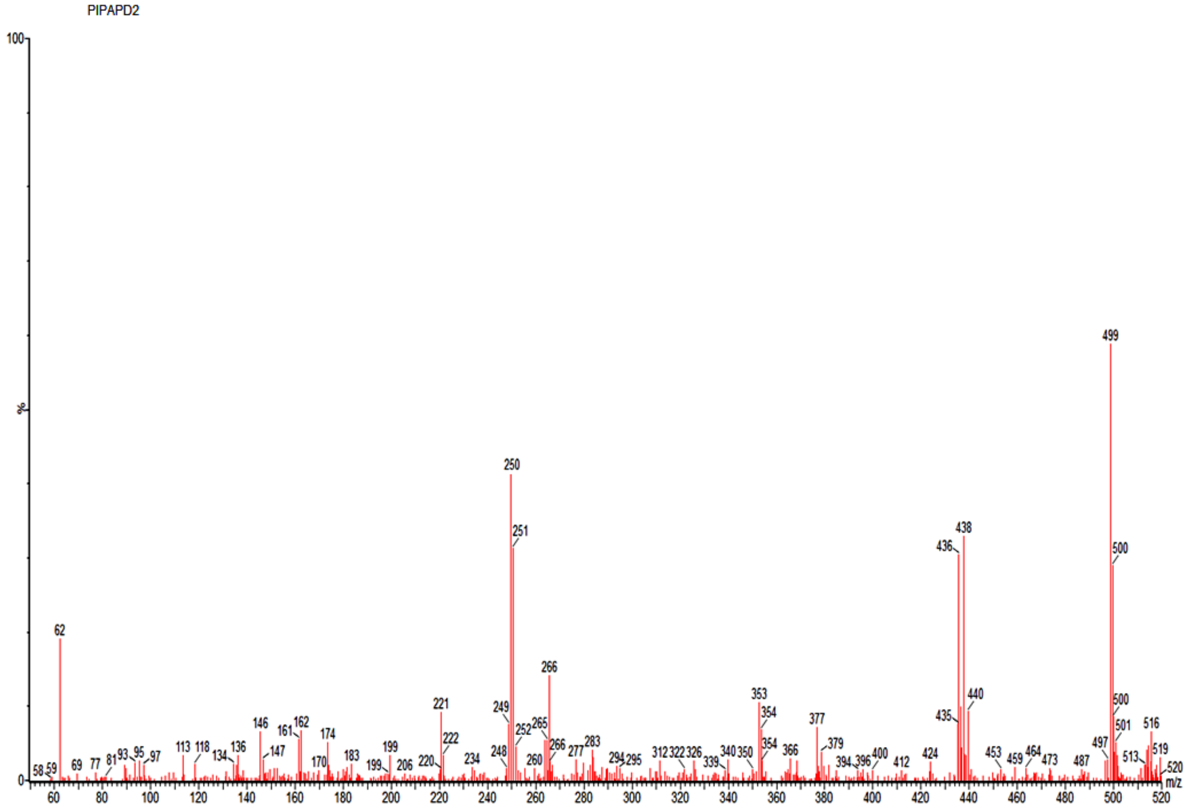

Figure S18. Mass spectrum of **6b**.

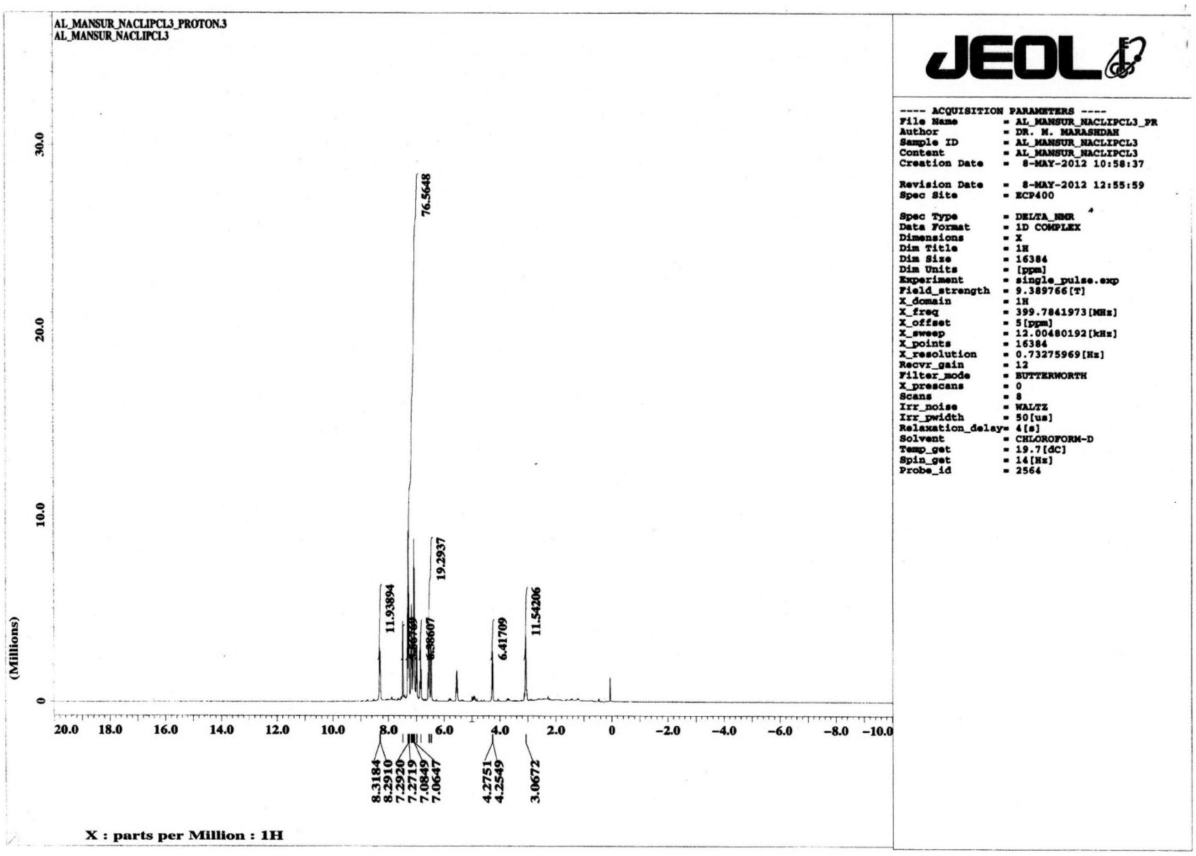

Figure S19. ^{1}H-NMR spectrum of **5c**.

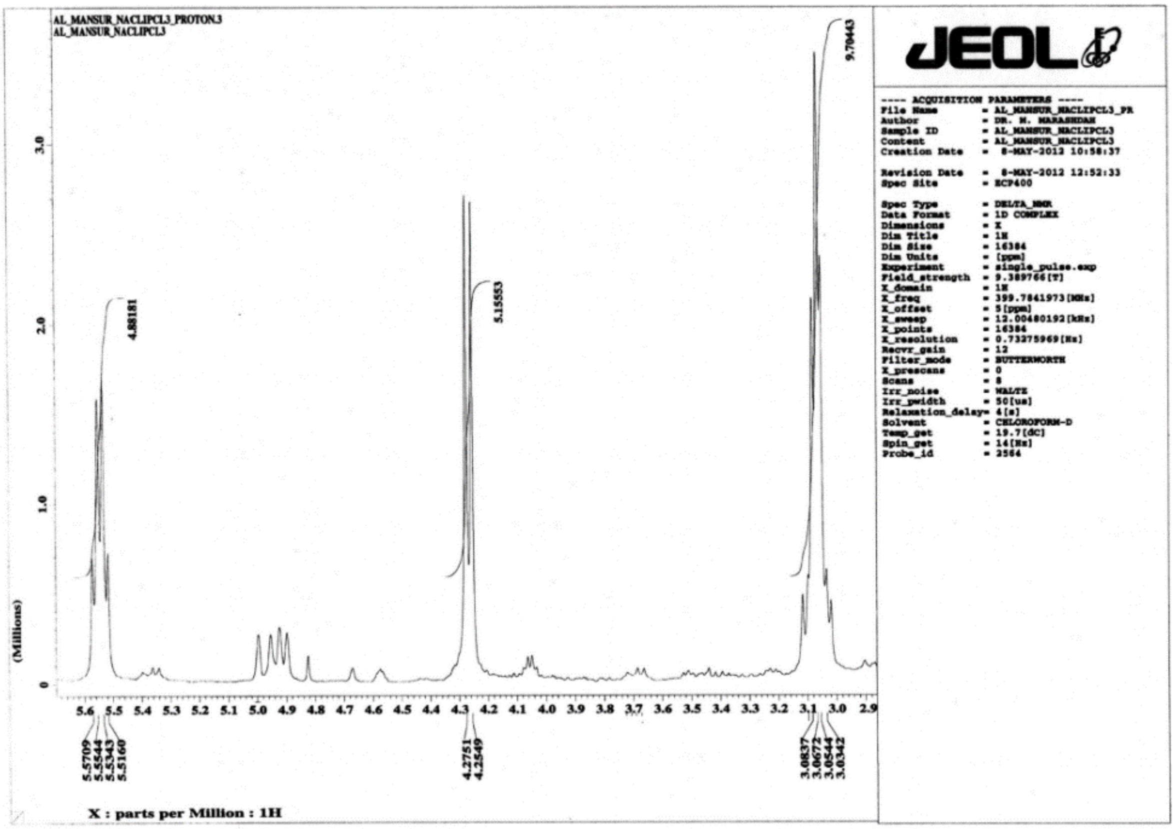

Figure S20. Expanded ^{1}H-NMR spectrum of **5c**.

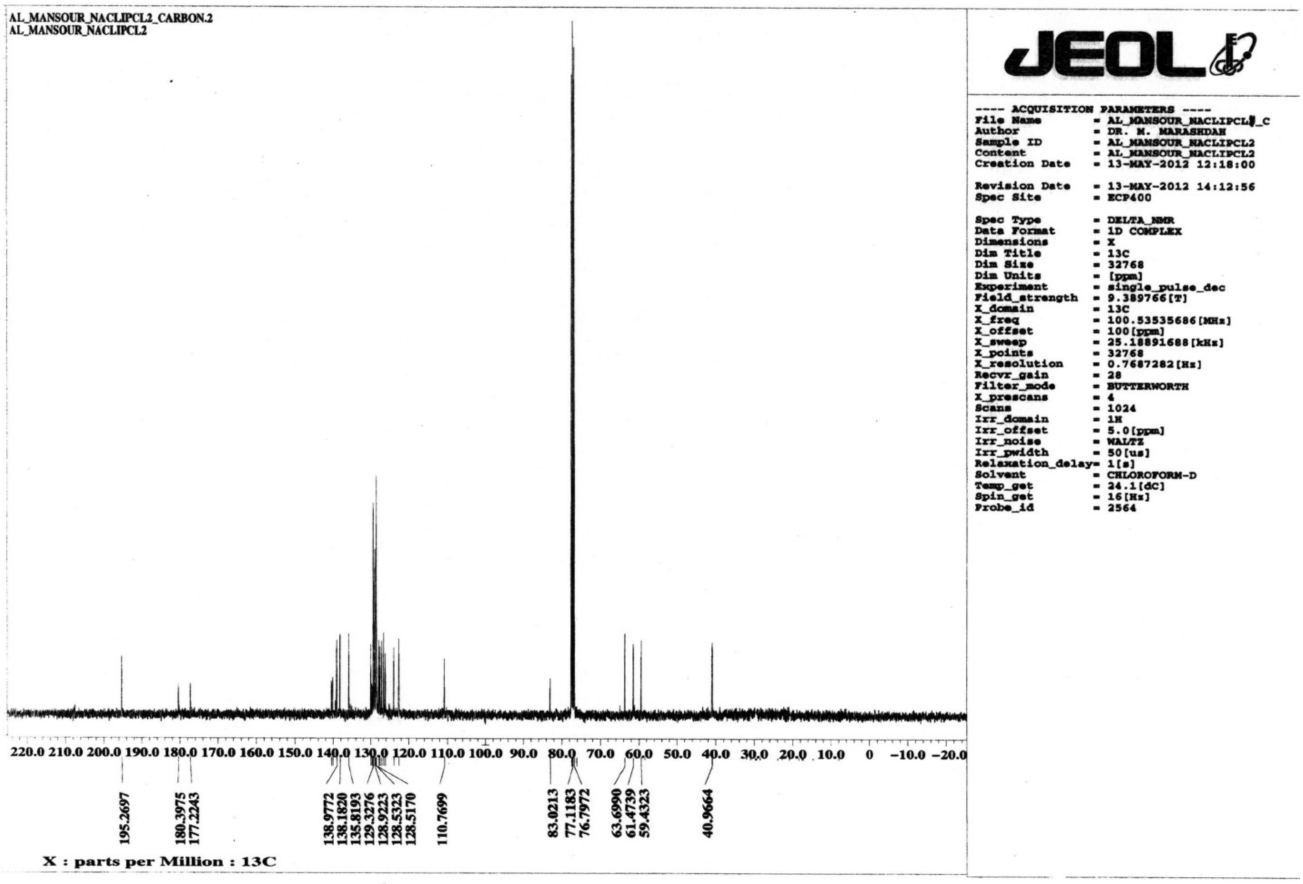

Figure S21. ^{13}C-NMR spectrum of **5c**.

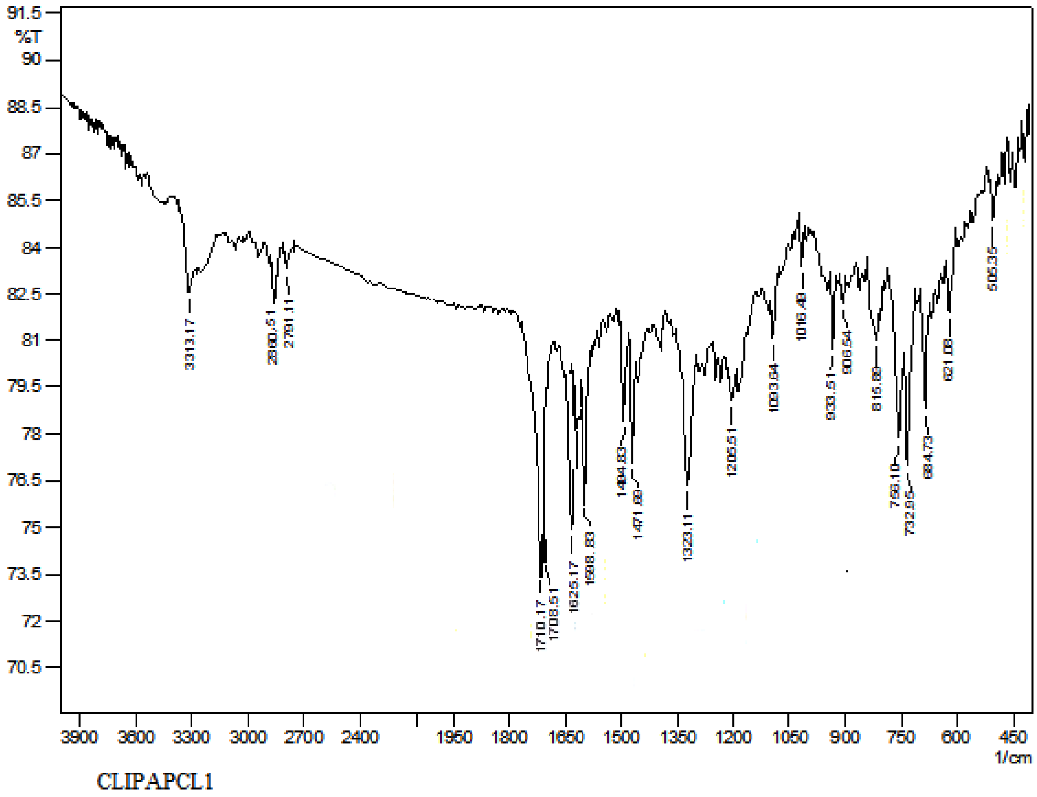

Figure S22. IR spectrum of **5c**.

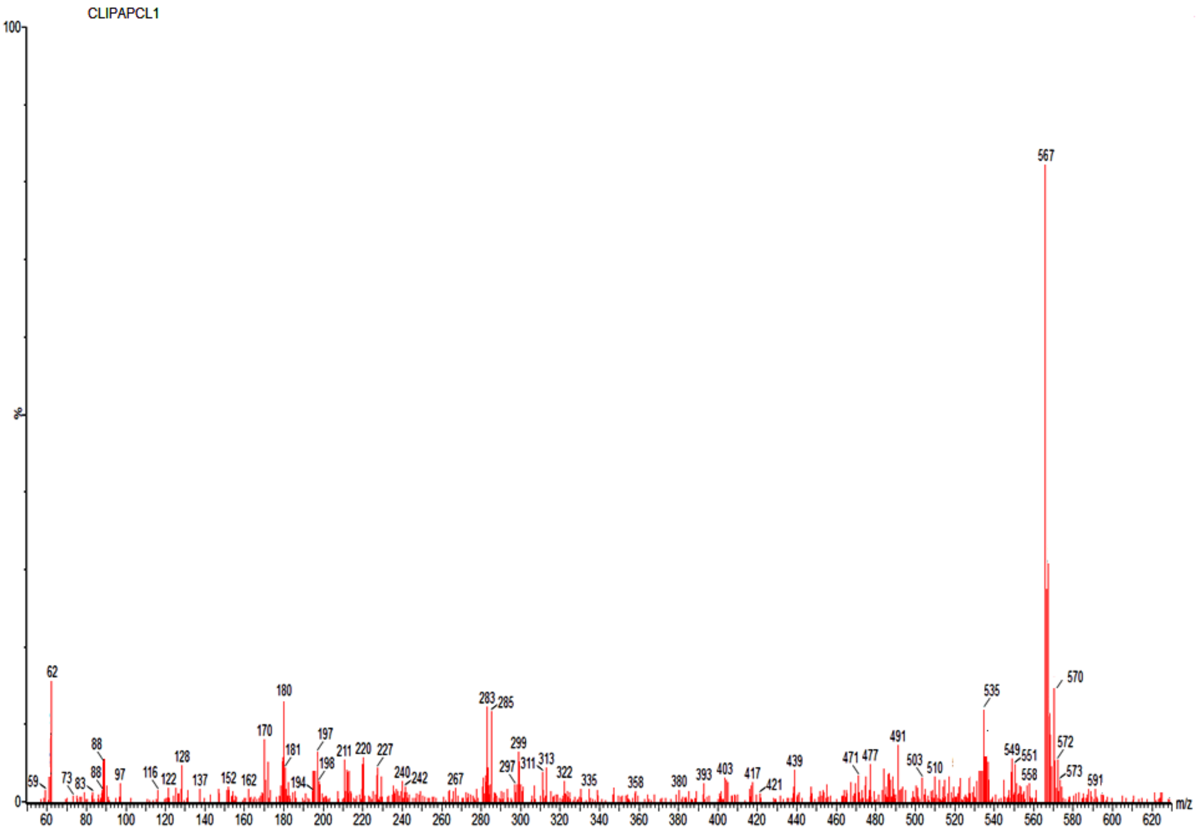

Figure S23. Mass spectrum of **5c**.

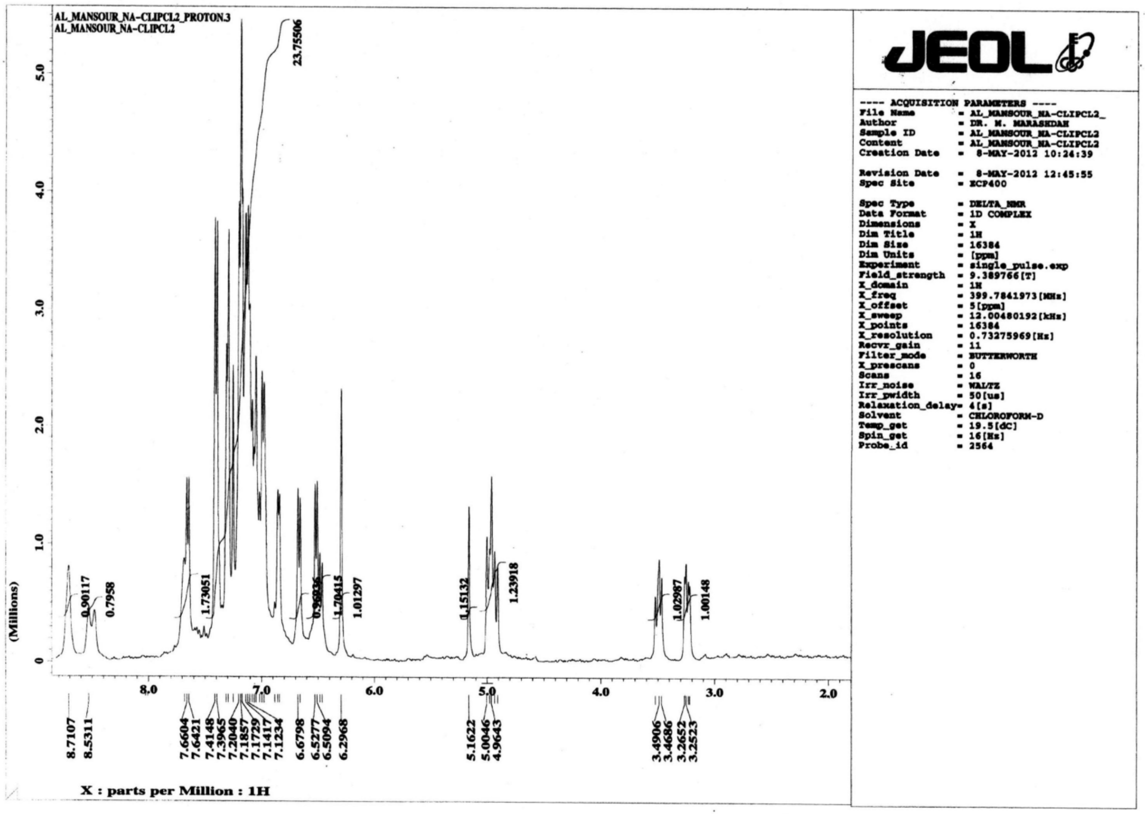

Figure S24. ^{1}H-NMR spectrum of **6c**.

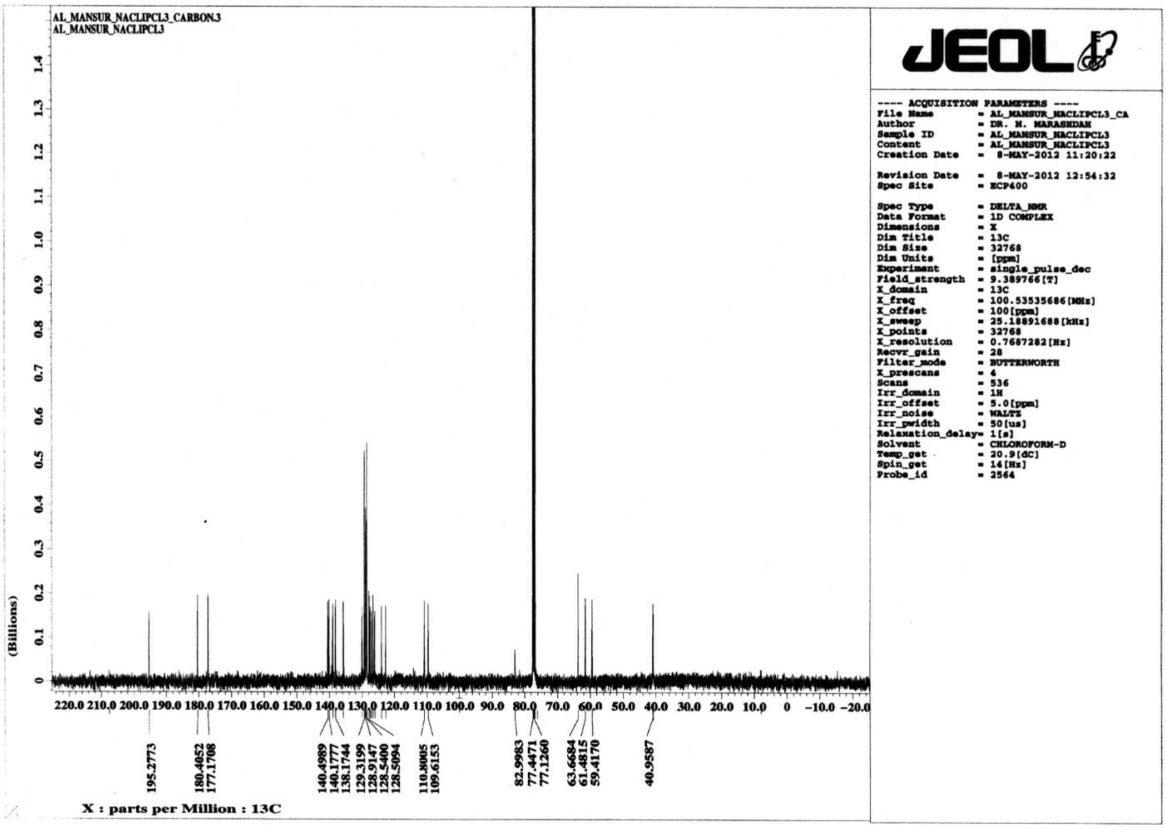

Figure S25. ^{13}C-NMR spectrum of **6c**.

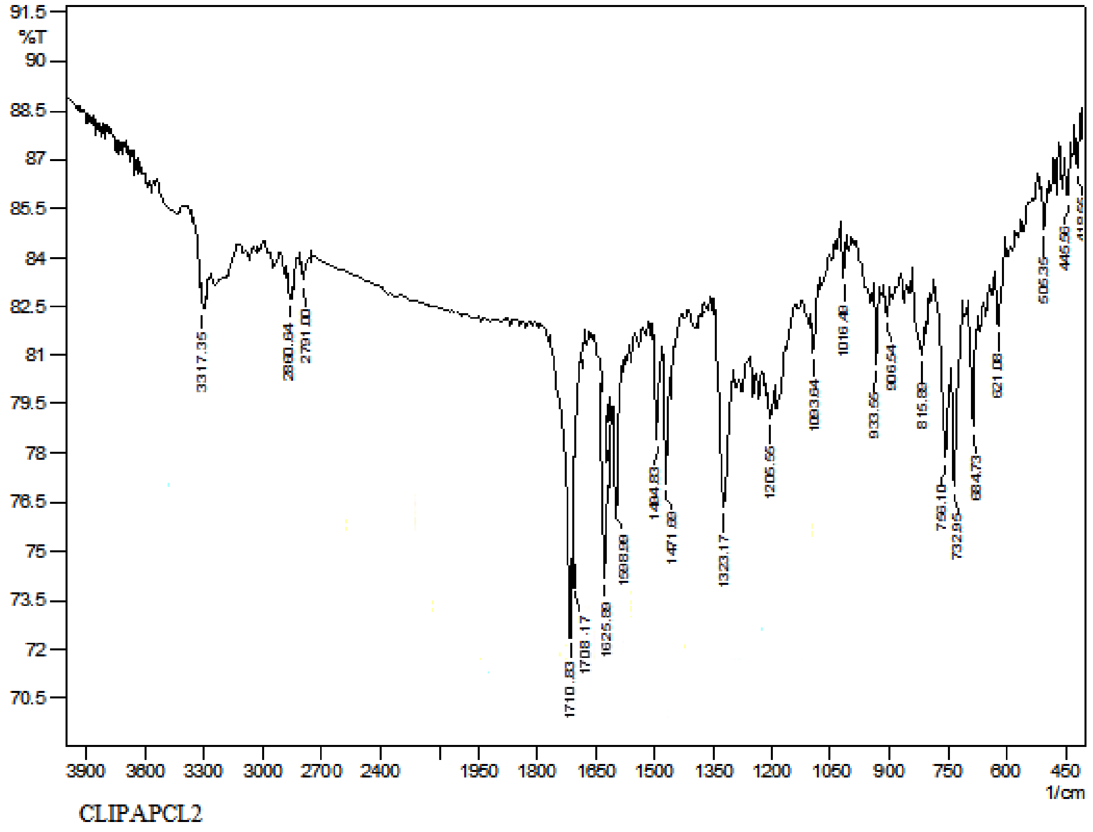

Figure S26. IR spectrum of **6c**.

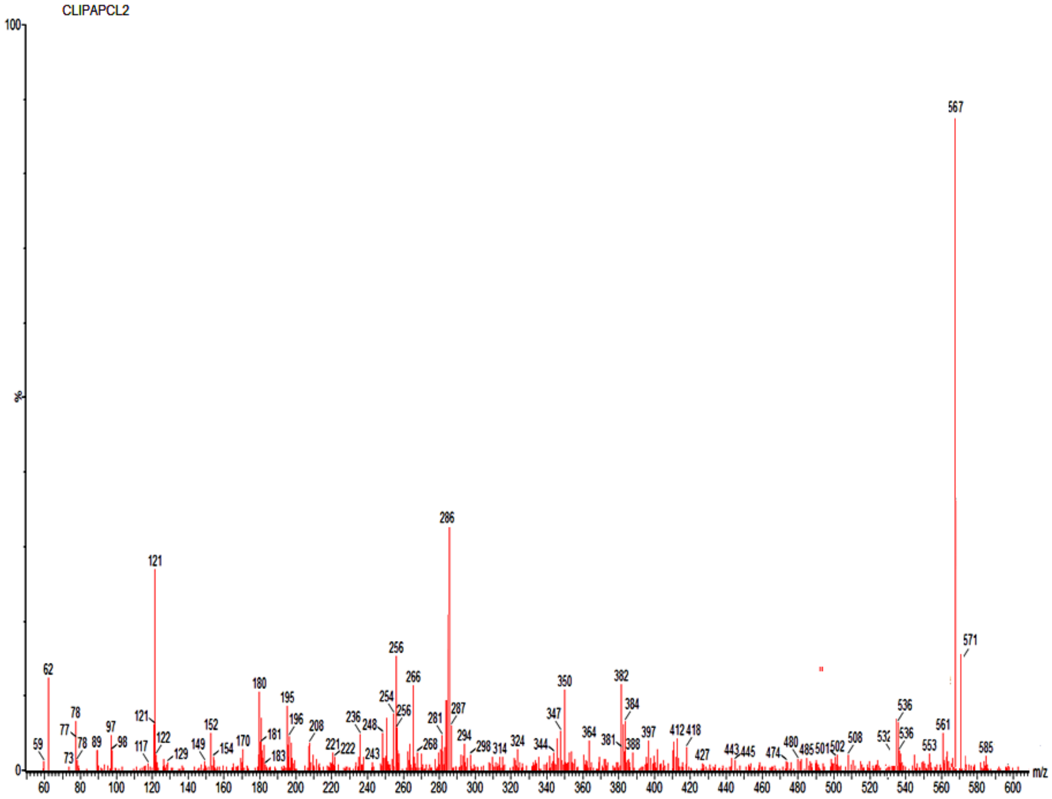

Figure S27. Mass spectrum of **6c**.

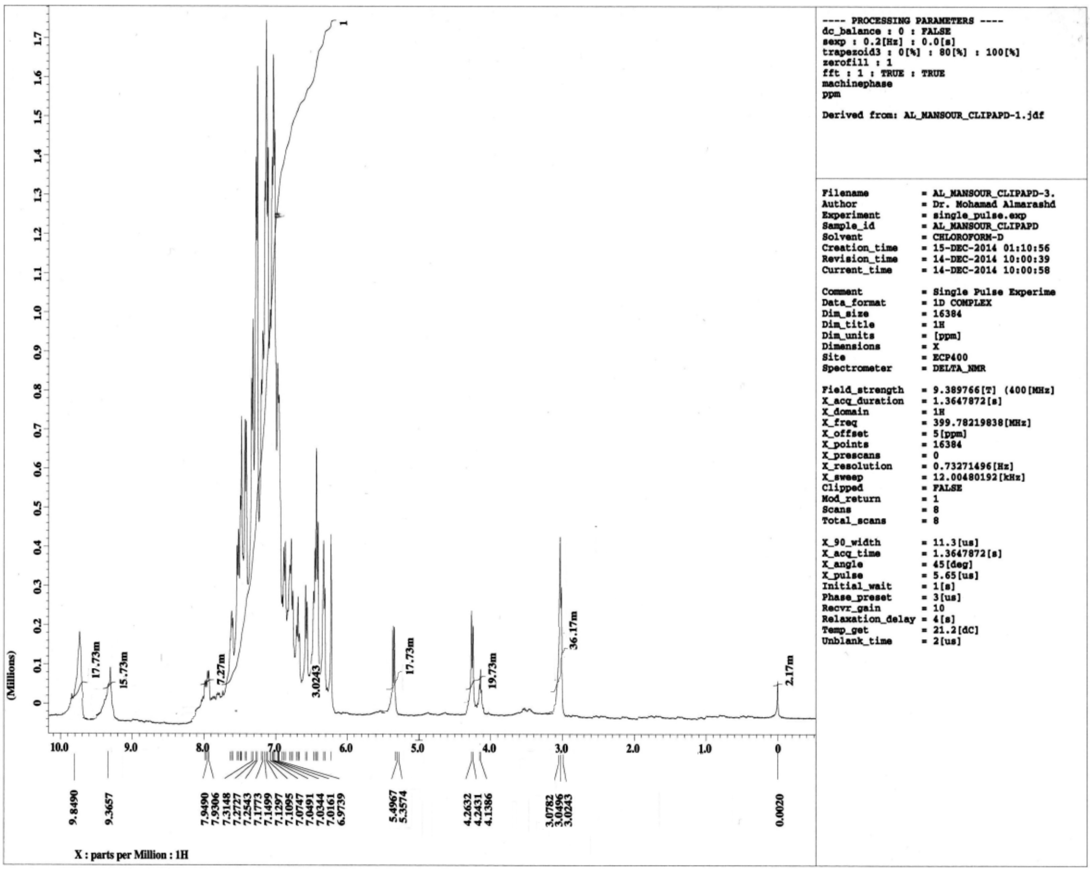

Figure S28. ^{1}H-NMR spectrum of **5d**.

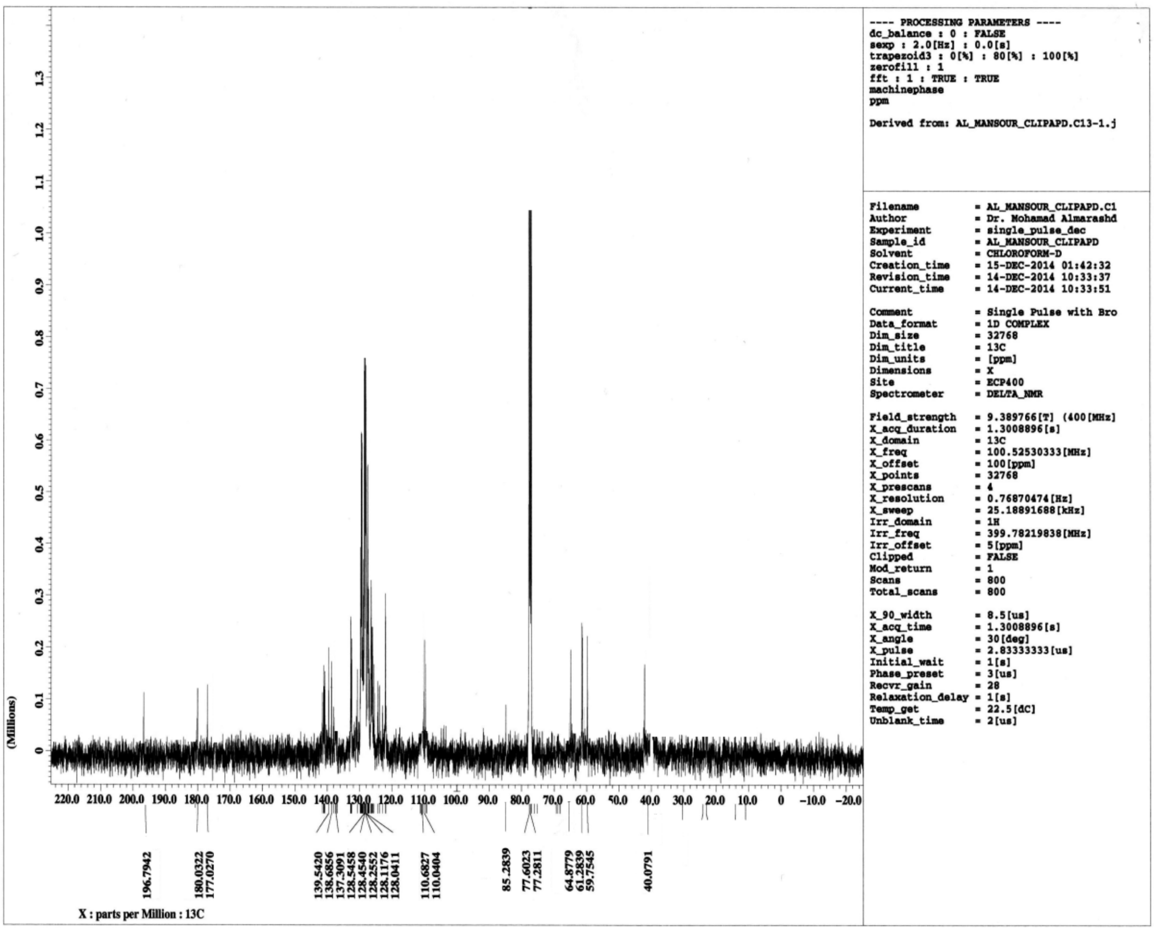

Figure S29. ^{13}C-NMR spectrum of **5d**.

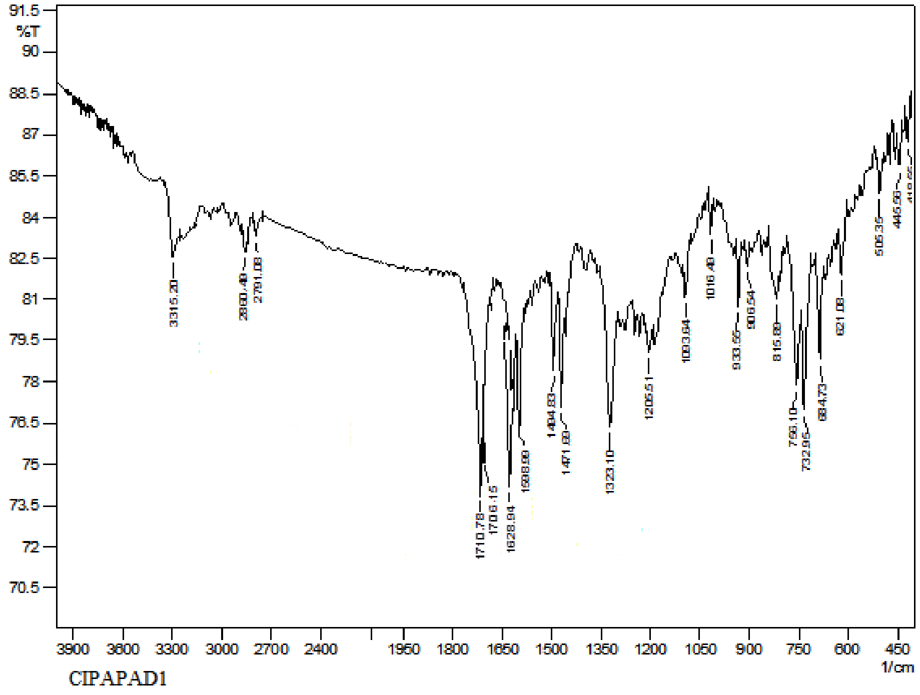

Figure S30. IR spectrum of **5d**.

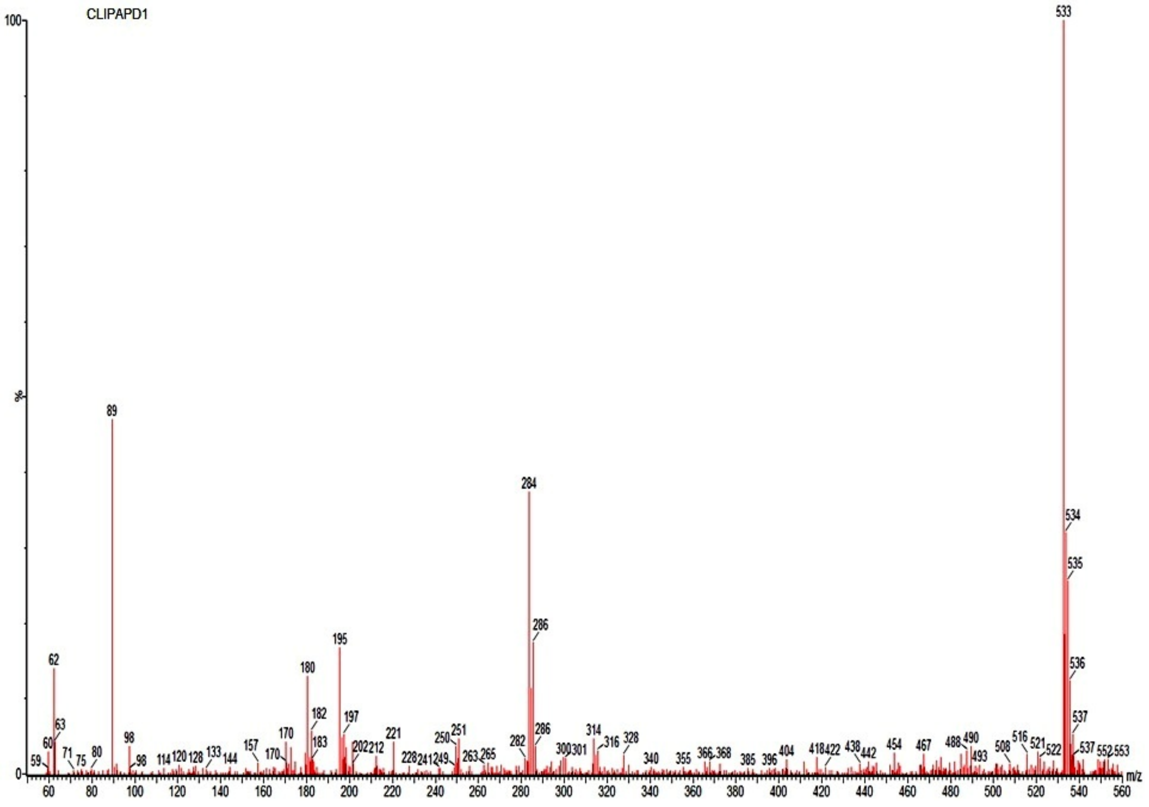

Figure S31. Mass spectrum of **5d**.

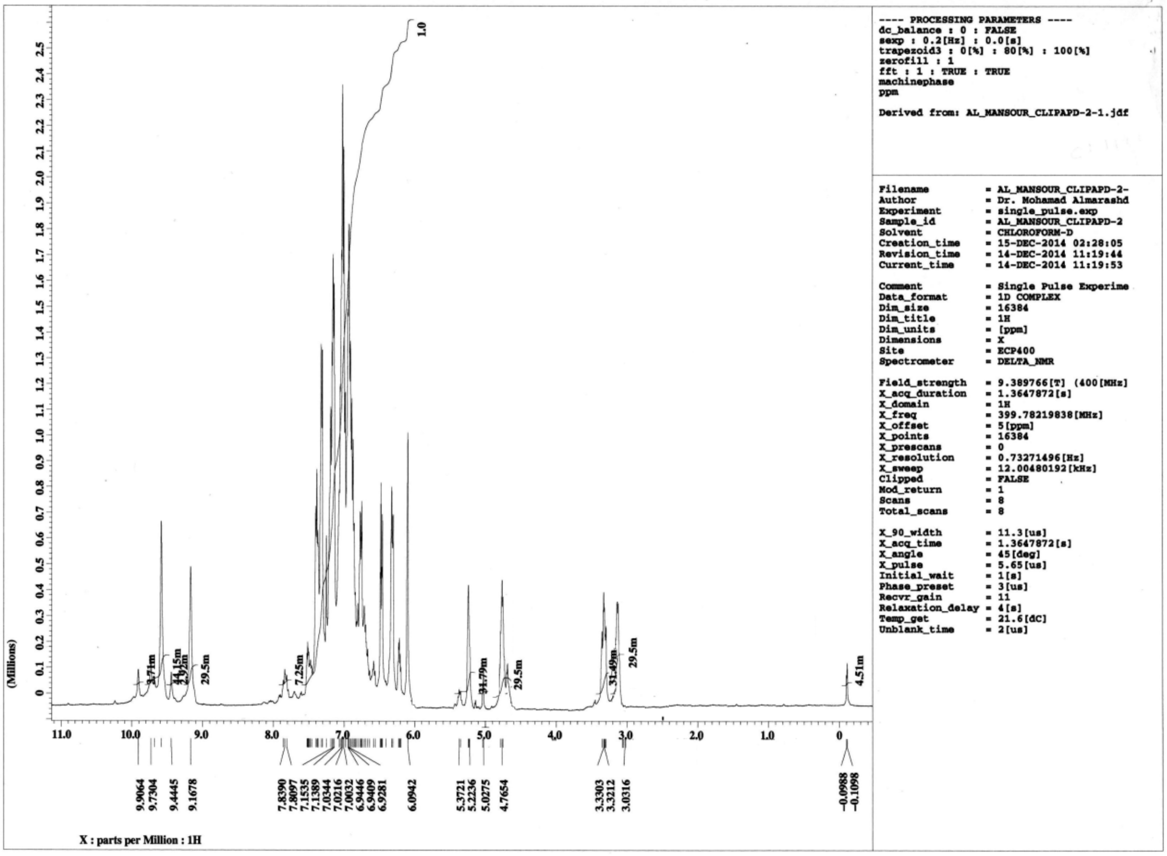

Figure S32. ^{1}H-NMR spectrum of **6d**.

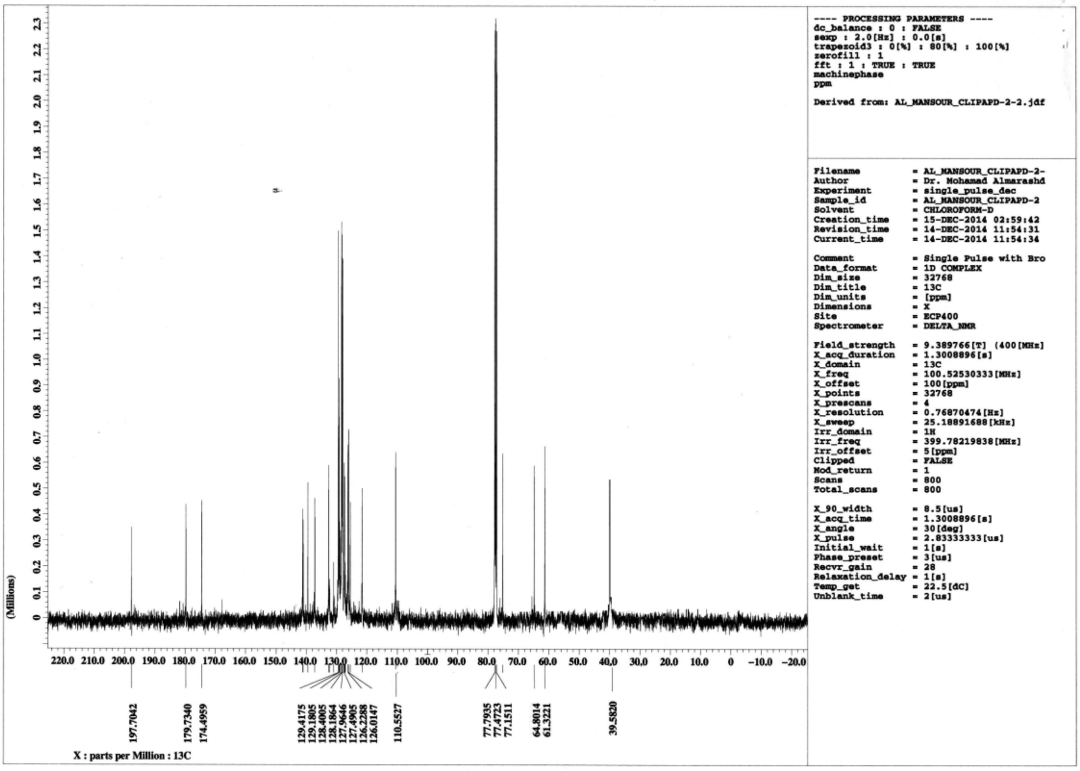

Figure S33. ^{13}C-NMR spectrum of **6d**.

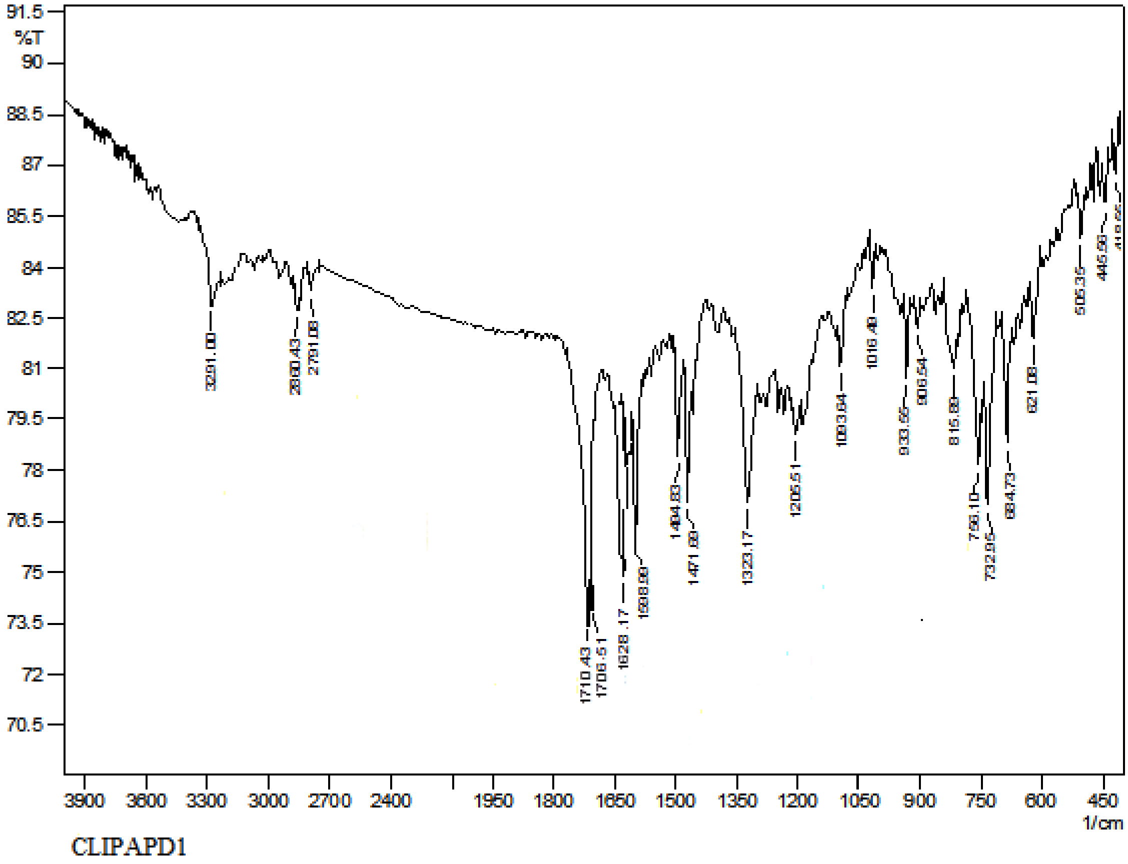

Figure S34. IR spectrum of **6d**.

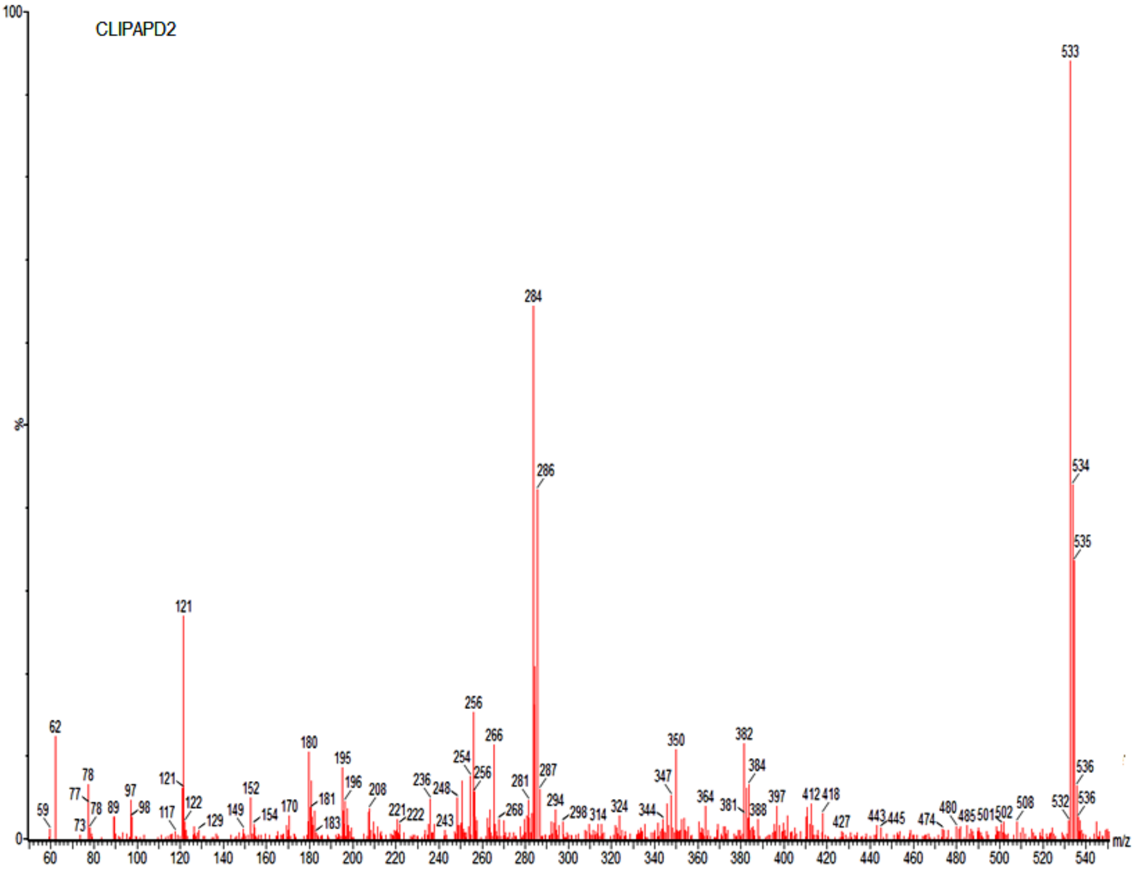

Figure S35. Mass spectrum of **6d**.

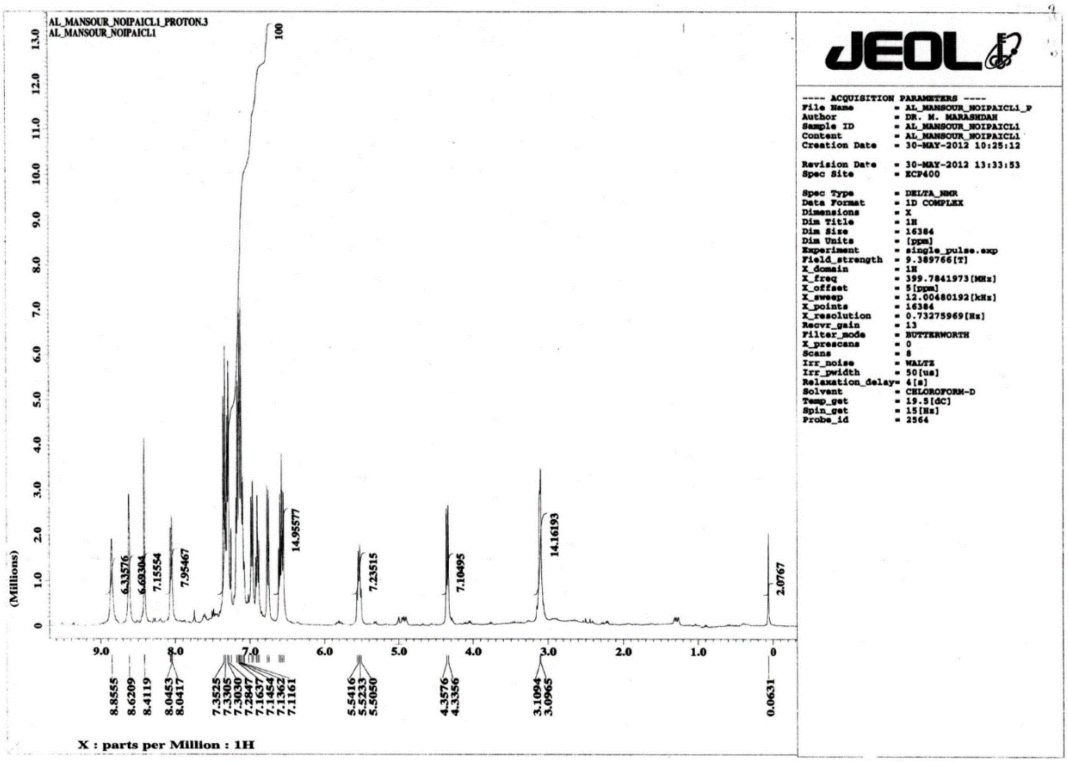

Figure S36. ^{1}H-NMR spectrum of **5e**.

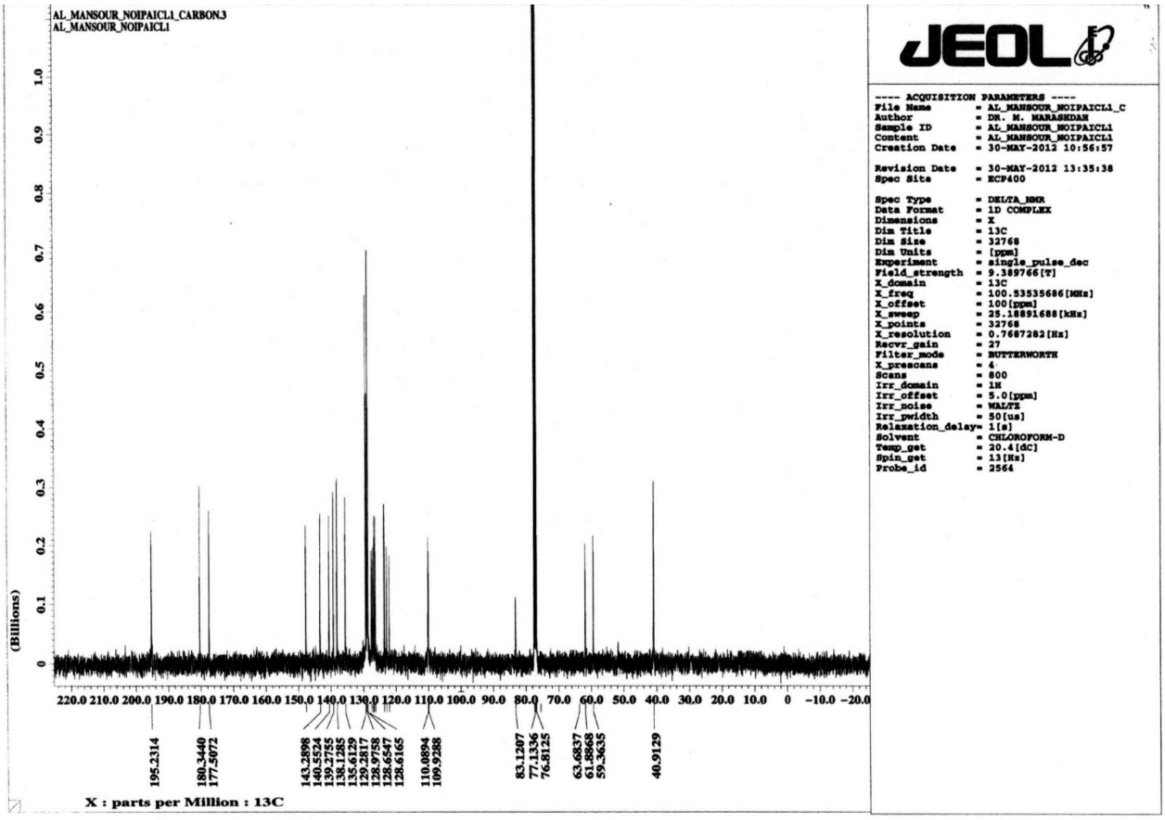

Figure S37. ^{13}C-NMR spectrum of **5e**.

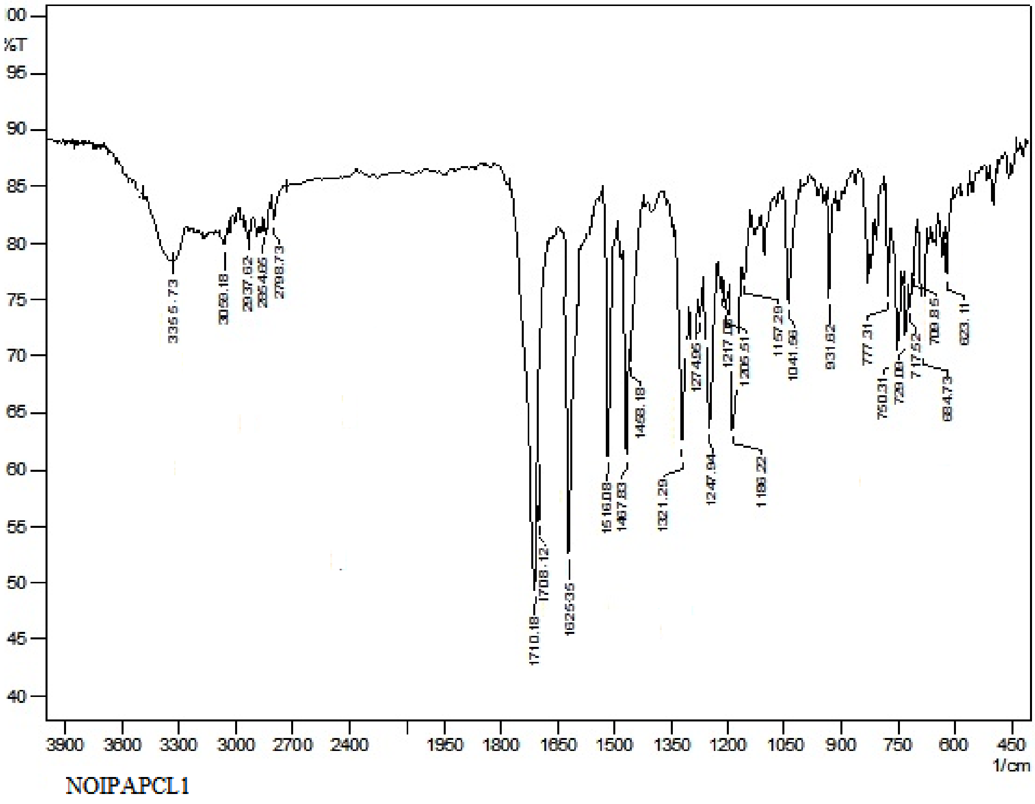

Figure S38. IR spectrum of **5e**.

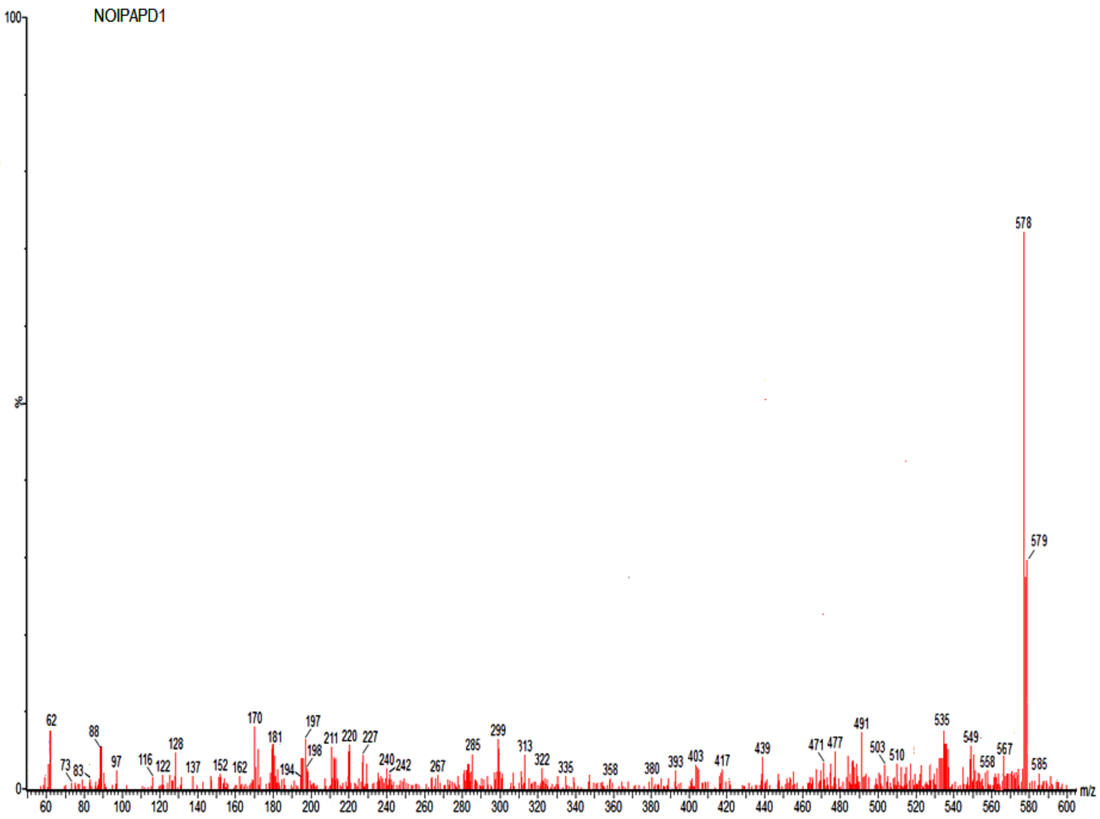

Figure S39. Mass spectrum of **5e**.

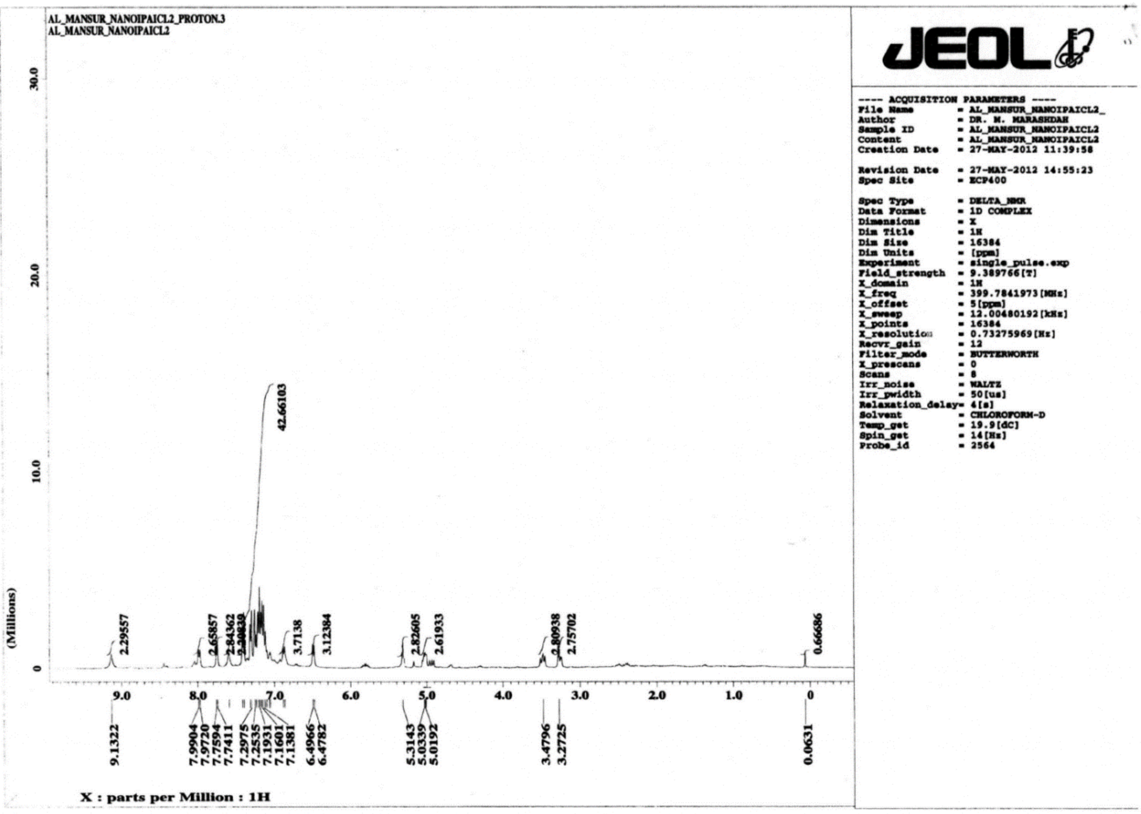

Figure S40. ^{1}H-NMR spectrum of **6e**.

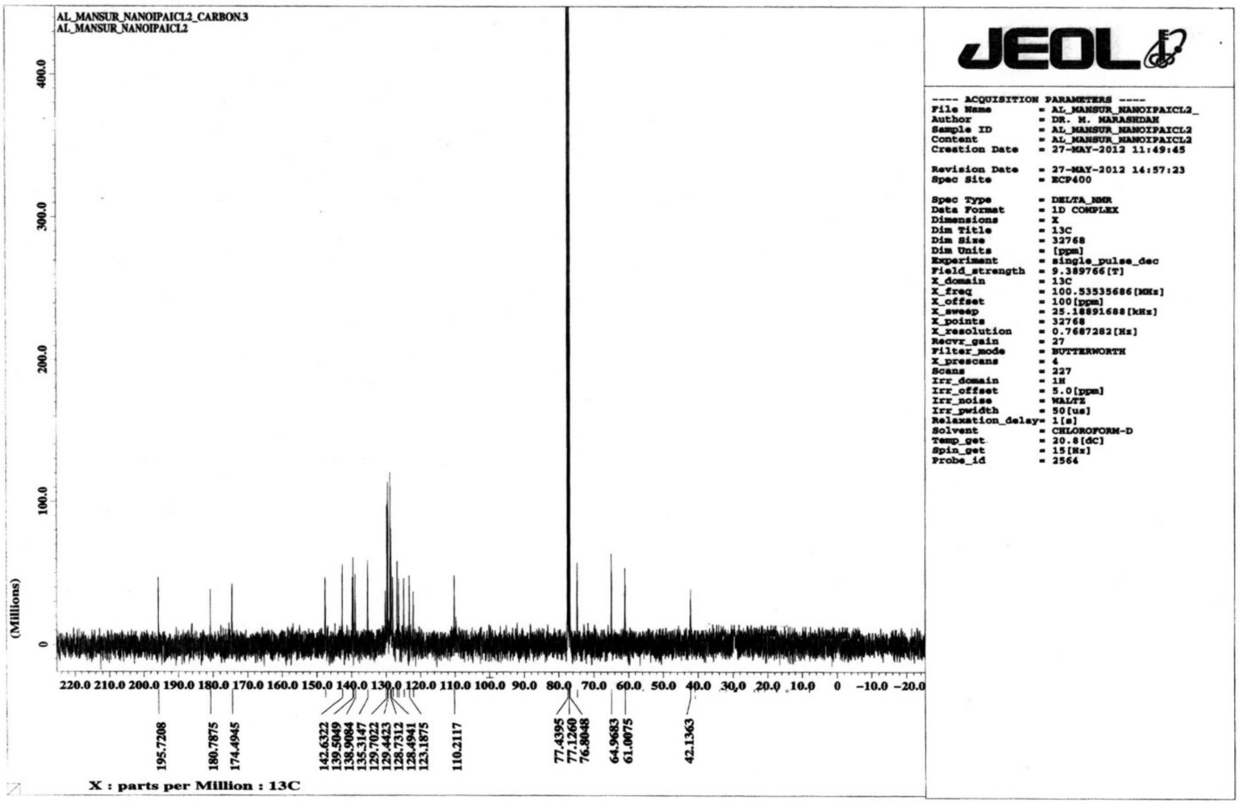

Figure S41. ^{13}C-NMR spectrum of **6e**.

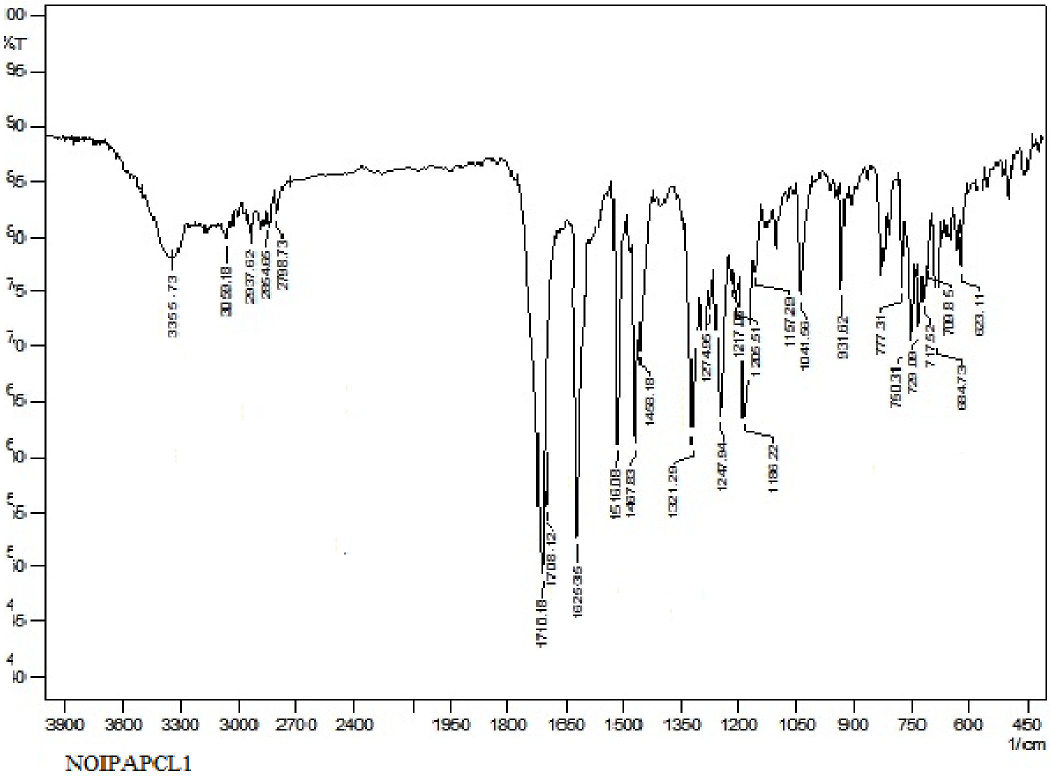

Figure S42. IR spectrum of **6e**.

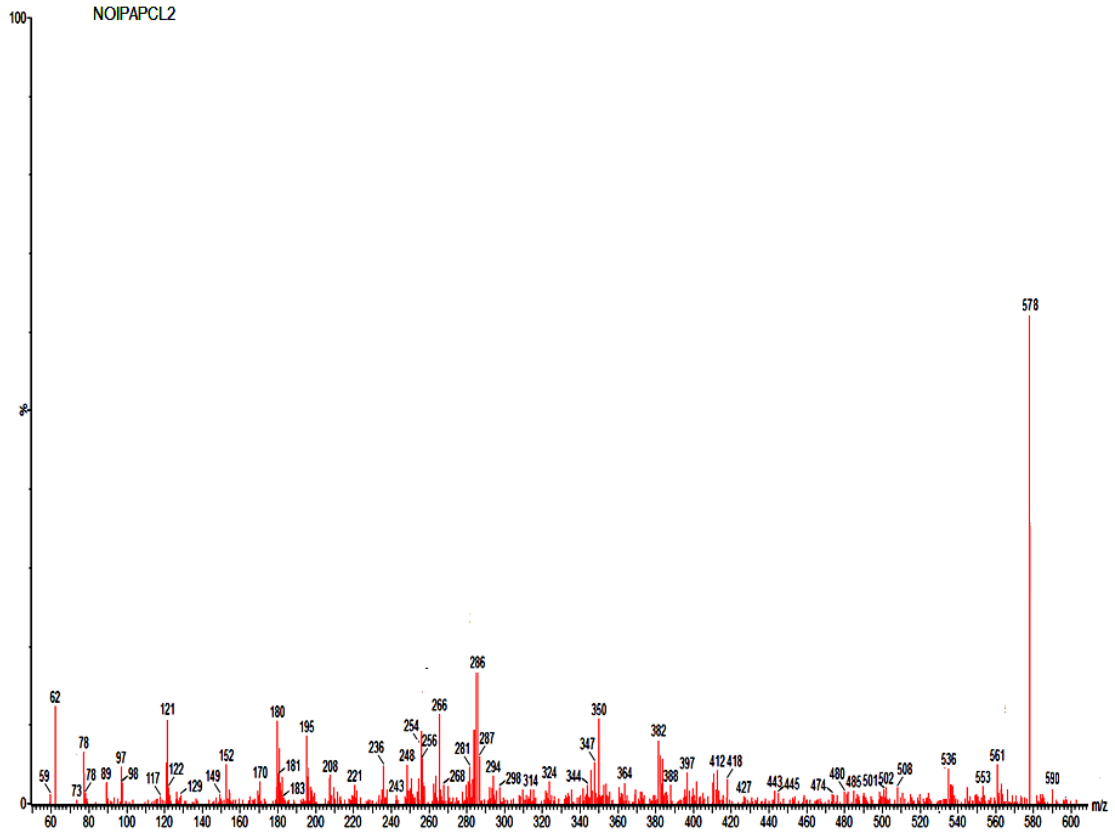

Figure S43. Mass spectrum of **6e**.

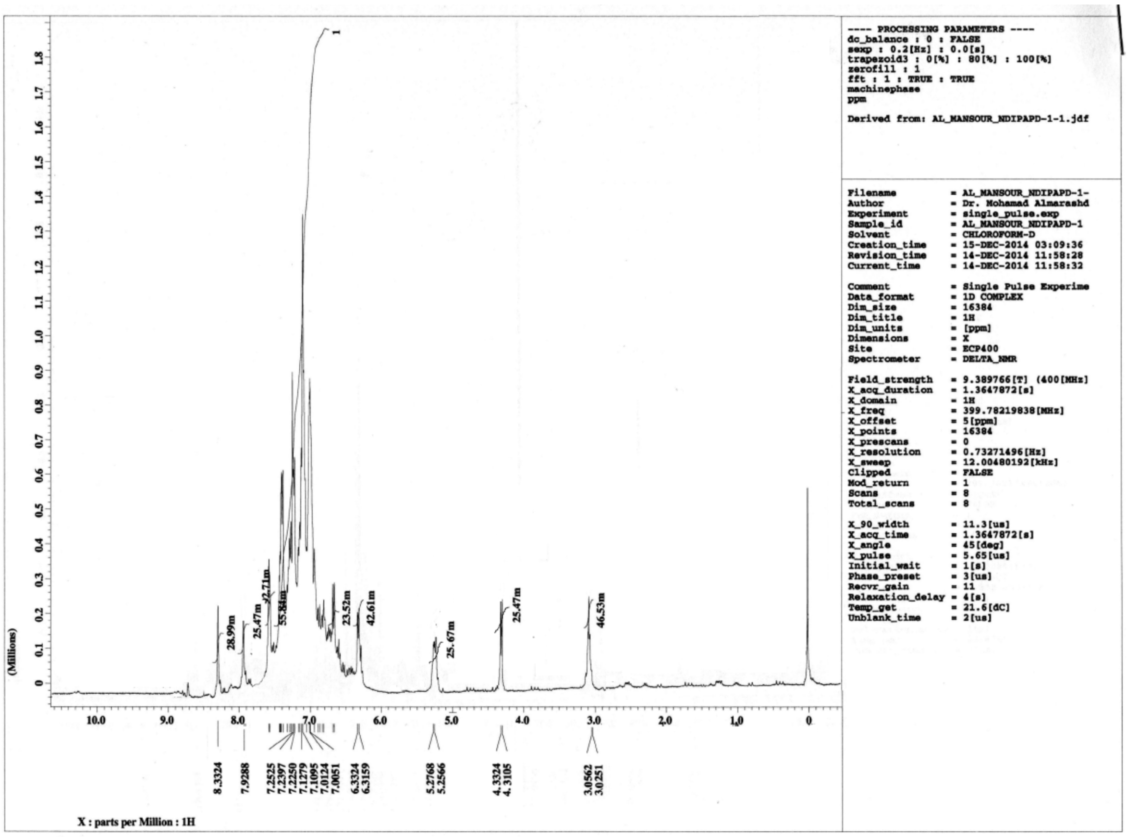

Figure S44. ^{1}H-NMR spectrum of **5f**.

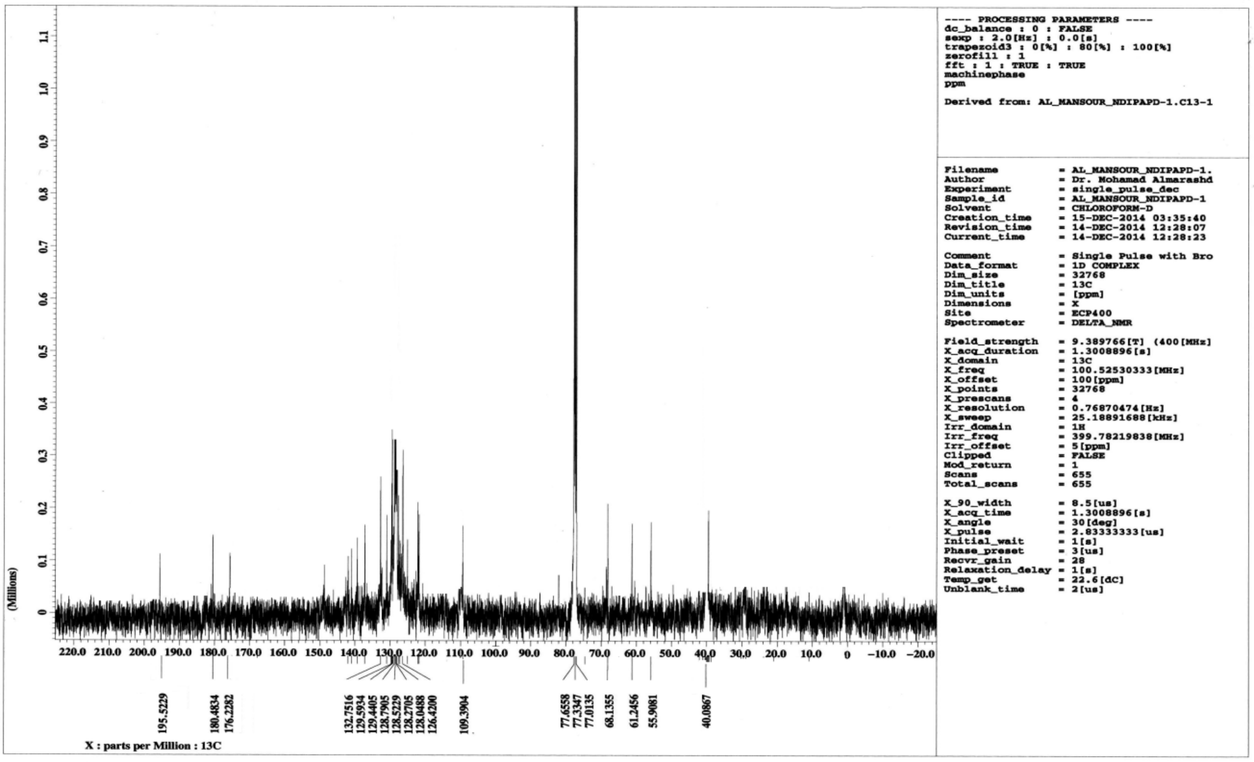

Figure S45. ^{13}C-NMR spectrum of **5f**.

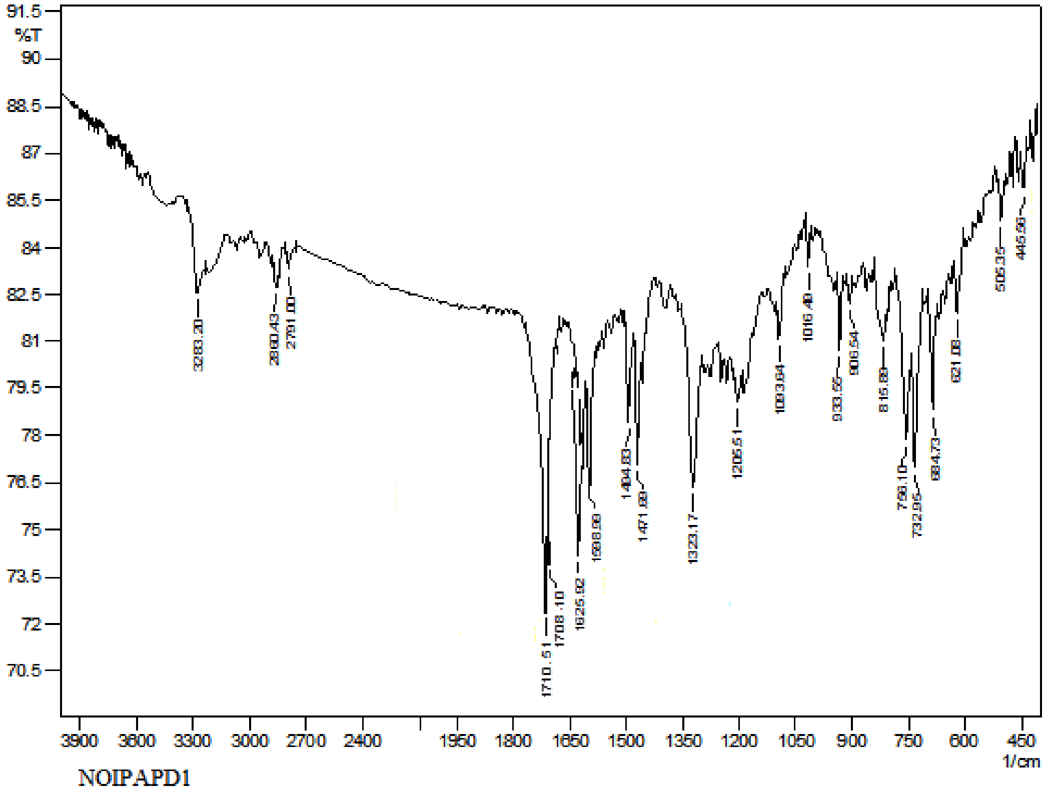

Figure S46. IR spectrum of **5f**.

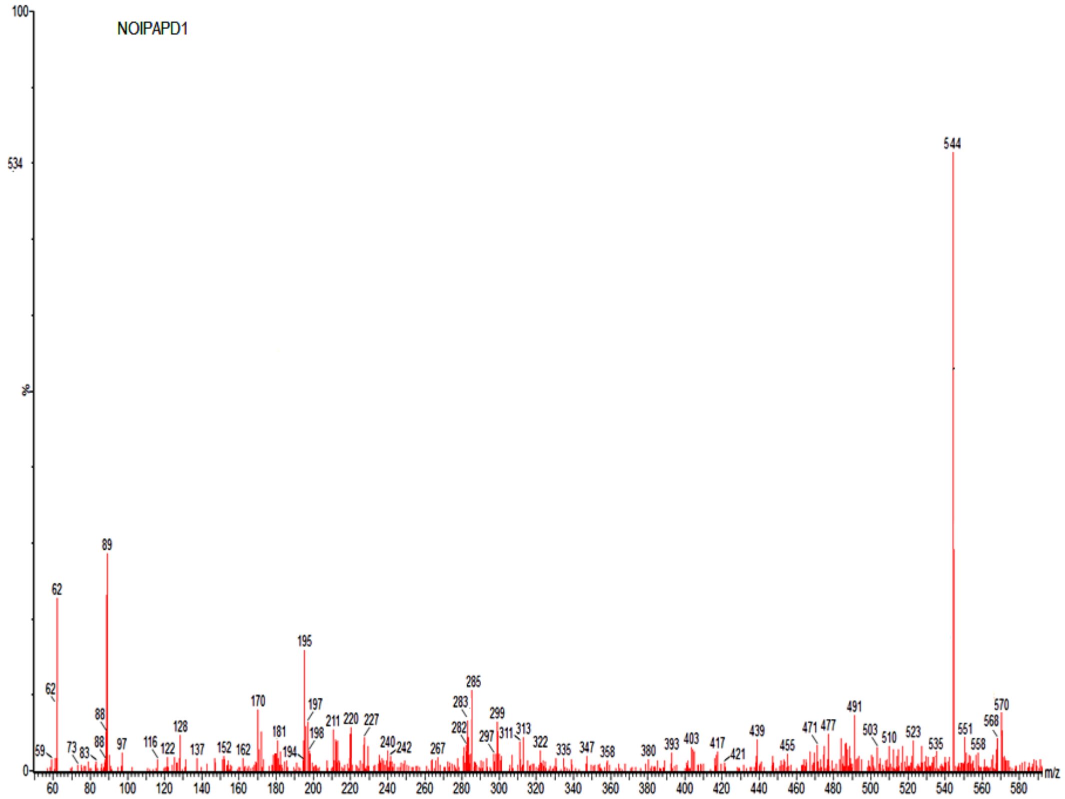

Figure S47. Mass spectrum of **5f**.

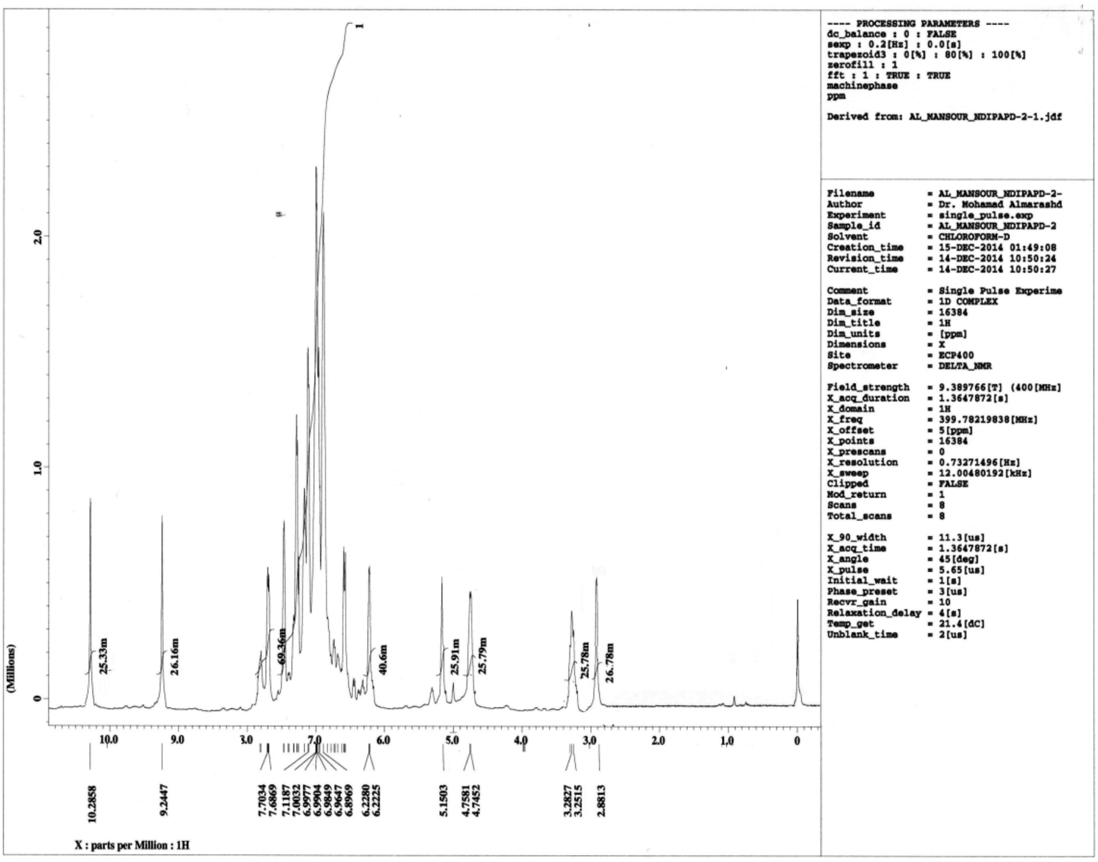

Figure S48. ^{1}H-NMR spectrum of **6f**.

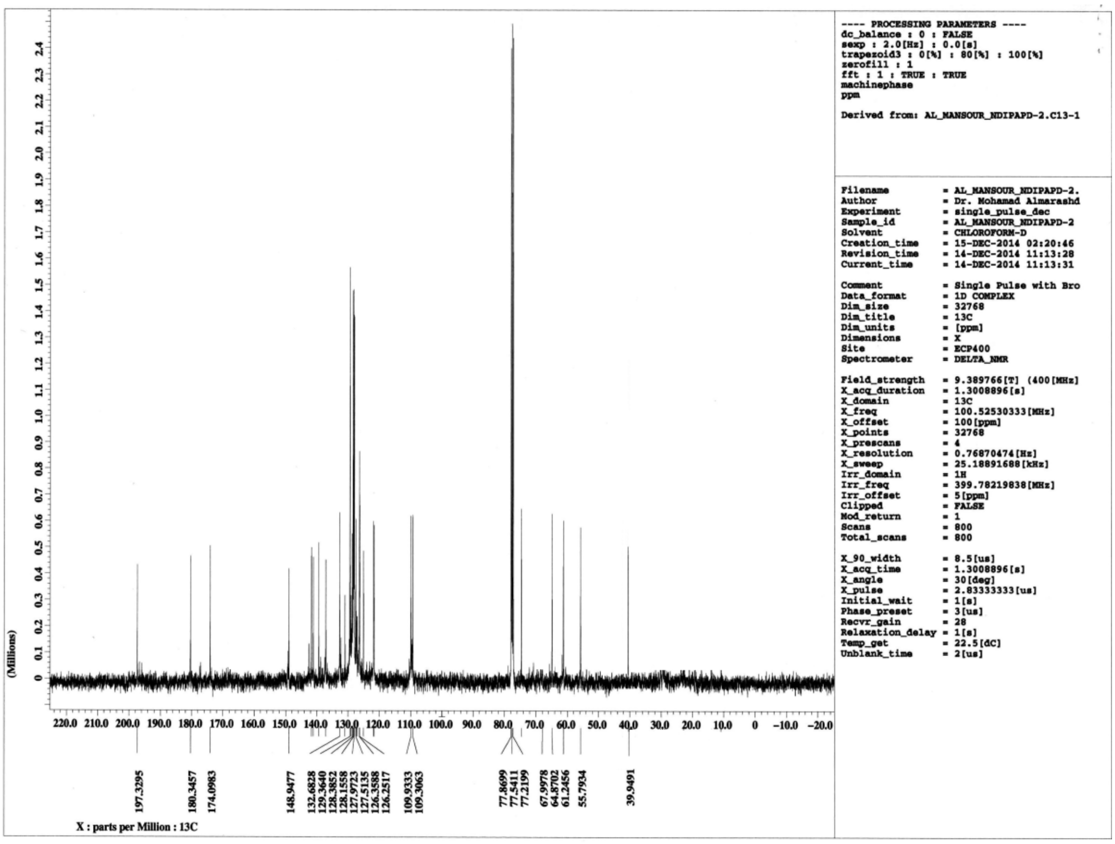

Figure S49. ^{13}C-NMR spectrum of **6f**.

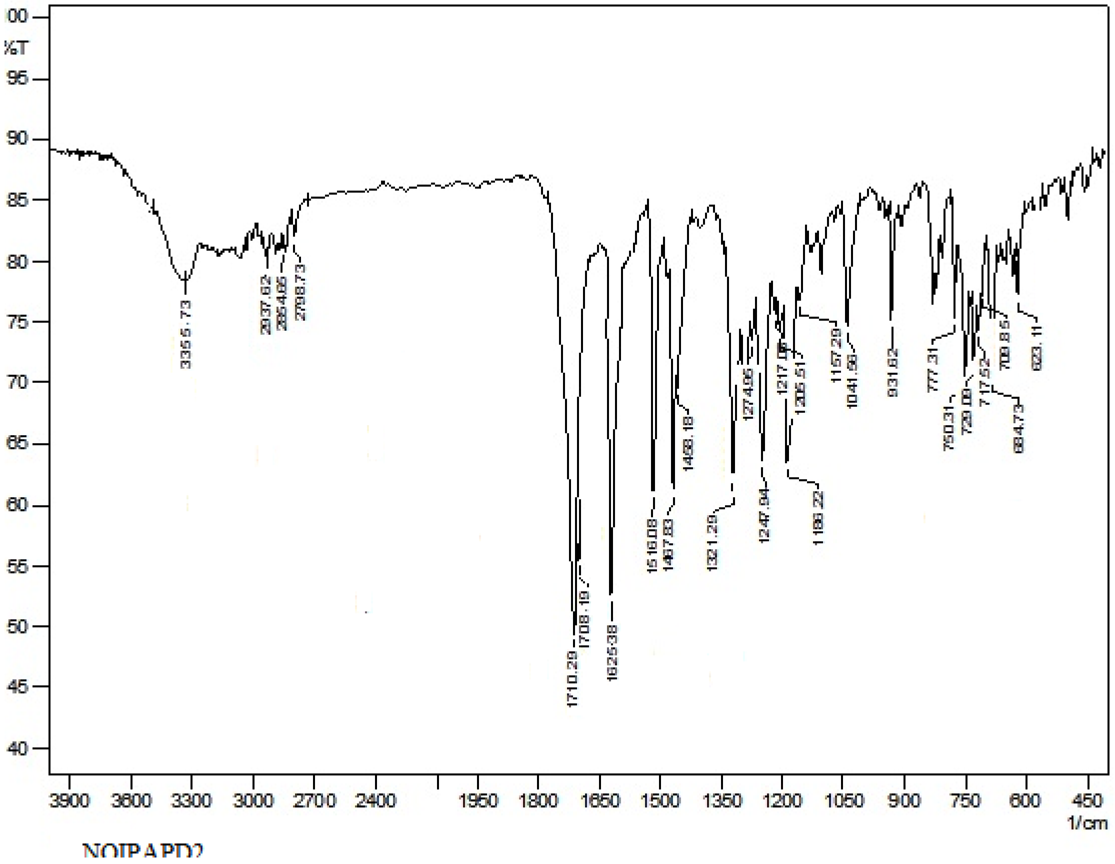

Figure S50. IR spectrum of **6f**.

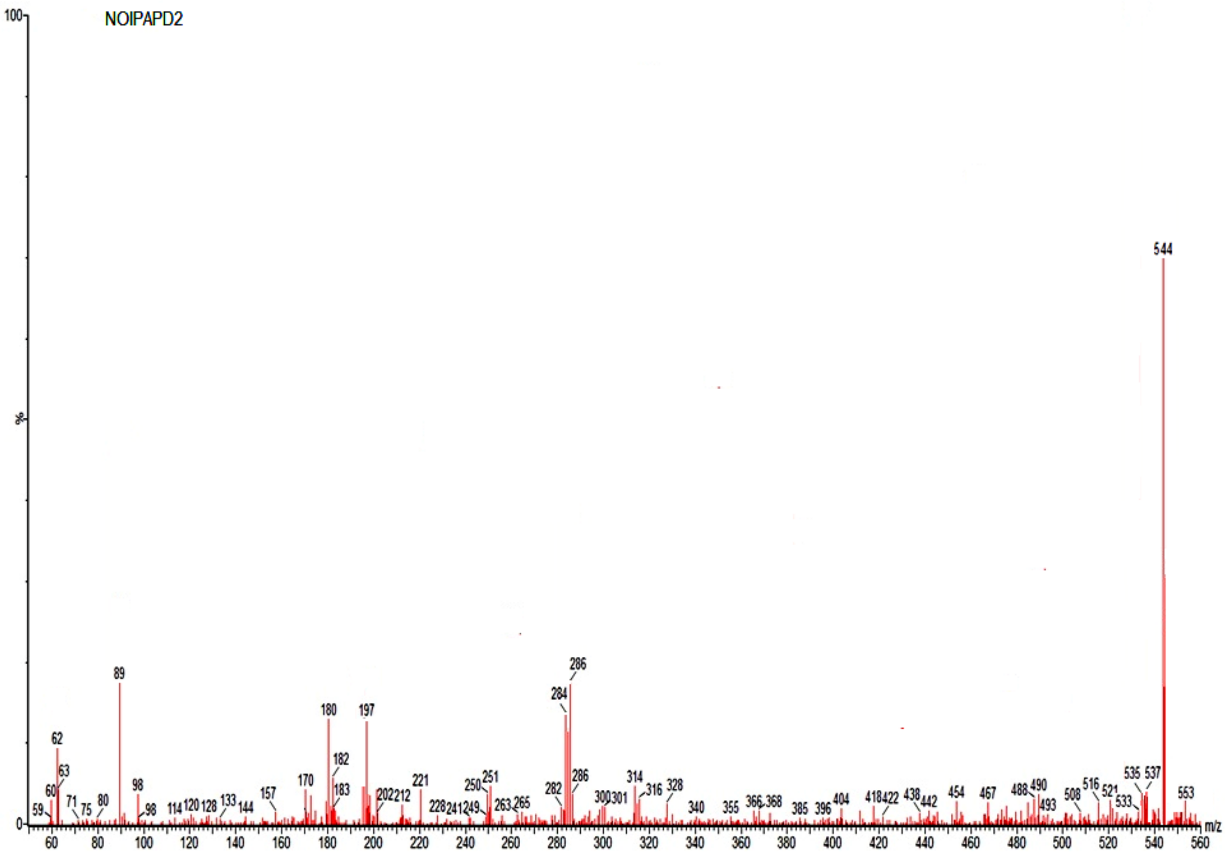

Figure S51. Mass spectrum of **6f**.
